# SARM1 promotes neuroinflammation and inhibits neural regeneration after spinal cord injury through NF-κB signaling

**DOI:** 10.7150/thno.49054

**Published:** 2021-02-20

**Authors:** Huitao Liu, Jingjing Zhang, Xingxing Xu, Sheng Lu, Danlu Yang, Changnan Xie, Mengxian Jia, Wenbin Zhang, Lingting Jin, Xiwu Wang, Xiya Shen, Fayi Li, Wangfei Wang, Xiaomei Bao, Sijia Li, Minyu Zhu, Wei Wang, Ying Wang, Zhihui Huang, Honglin Teng

**Affiliations:** 1 Department of Orthopedics (Spine Surgery), The First Affiliated Hospital of Wenzhou Medical University, Wenzhou 325000, Zhejiang, China.; 2 School of Basic Medical Sciences, Wenzhou Medical University, Wenzhou 325035, Zhejiang, China.; 3 Department of Orthopedics, Taizhou Hospital of Zhejiang Province, Linhai 317000, Zhejiang, China.; 4 Engineering Laboratory of Development and Application of Traditional Chinese Medicine from Zhejiang Province and Holistic Integrative Pharmacy Institutes, Hangzhou Normal University, Hangzhou 311121, Zhejiang, China.; 5 School of Mental Health, Wenzhou Medical University, Wenzhou 325035, Zhejiang, China.; 6 Phase I Clinical Research Center, Zhejiang Provincial People's Hospital of Hangzhou Medical College, Hangzhou, Zhejiang, 310053, China.

**Keywords:** SARM1, spinal cord injury, neuroinflammation, neural regeneration, axonal degeneration

## Abstract

Axonal degeneration is a common pathological feature in many acute and chronic neurological diseases such as spinal cord injury (SCI). SARM1 (sterile alpha and TIR motif-containing 1), the fifth TLR (Toll-like receptor) adaptor, has diverse functions in the immune and nervous systems, and recently has been identified as a key mediator of Wallerian degeneration (WD). However, the detailed functions of SARM1 after SCI still remain unclear.

**Methods:** Modified Allen's method was used to establish a contusion model of SCI in mice. Furthermore, to address the function of SARM1 after SCI, conditional knockout (CKO) mice in the central nervous system (CNS), SARM1^Nestin^-CKO mice, and SARM1^GFAP^-CKO mice were successfully generated by Nestin-Cre and GFAP-Cre transgenic mice crossed with SARM1^flox/flox^ mice, respectively. Immunostaining, Hematoxylin-Eosin (HE) staining, Nissl staining and behavioral test assays such as footprint and Basso Mouse Scale (BMS) scoring were used to examine the roles of SARM1 pathway in SCI based on these conditional knockout mice. Drugs such as FK866, an inhibitor of SARM1, and apoptozole, an inhibitor of heat shock protein 70 (HSP70), were used to further explore the molecular mechanism of SARM1 in neural regeneration after SCI.

**Results:** We found that SARM1 was upregulated in neurons and astrocytes at early stage after SCI. SARM1^Nestin^-CKO and SARM1^GFAP^-CKO mice displayed normal development of the spinal cords and motor function. Interestingly, conditional deletion of SARM1 in neurons and astrocytes promoted the functional recovery of behavior performance after SCI. Mechanistically, conditional deletion of SARM1 in neurons and astrocytes promoted neuronal regeneration at intermediate phase after SCI, and reduced neuroinflammation at SCI early phase through downregulation of NF-κB signaling after SCI, which may be due to upregulation of HSP70. Finally, FK866, an inhibitor of SARM1, reduced the neuroinflammation and promoted the neuronal regeneration after SCI.

**Conclusion:** Our results indicate that SARM1-mediated prodegenerative pathway and neuroinflammation promotes the pathological progress of SCI and anti-SARM1 therapeutics are viable and promising approaches for preserving neuronal function after SCI.

## Introduction

Spinal cord injury (SCI) is a severe central nervous system (CNS) damage resulting in a motor and sensory dysfunction, which may cause a permanent paralysis 1. The therapies of SCI mainly include surgeries, medications and rehabilitation treatments, however, clinical improvements in patients are still limited and unsatisfactory 2. This is largely due to the unique pathophysiology of SCI with the primary injury followed by a progressive secondary injury 1. In general, the primary injury is the result of physical forces including compression, shearing, laceration and acute stretch/distraction that induced by the initial traumatic events, and often determines the injury severity of SCI. The primary injury is followed by a cascade of secondary injury events that characterized by multiple injury processes including inflammation, glutamate excitotoxicity, apoptosis and free radical-induced cell death, and serves to expand the zone of injured neural tissue and exacerbates neurological deficits and outcomes 3. The inflammatory responses including the activation of resident microglia, recruitment of macrophages, neutrophils and lymphocytes from the bloodstream to the injury site, lead to profound neuropathological consequences such as neuronal death as well as axonal degeneration and demyelination, and the formation of glial scars 4. Although extensive studies have focused on the pathophysiological mechanism of SCI and it has been long recognized that axonal degeneration represents a key pathological feature, the pathogenesis of SCI and the intriguing links between axonal degeneration, neuroinflammation, synaptic growth and cell death after SCI still remain unclear.

A number of studies carried out in animals and human beings suggest that after SCI, the distal stumps of the long injury axons are irreversibly damaged and undergo a degenerative process, which is called Wallerian degeneration (WD), while the proximal stumps retract 5, 6. WD is a form of programmed self-destruction process that promotes axon breakdown in neurodegenerative diseases and axonal injury 7. SARM1 is a recently identified key mediator of WD 8, 9. SARM1 belongs to the TLR adaptor family, and encodes a protein with domains of sterile alpha motif (SAM) and armadillo motif (ARM), thus named SARM and renamed as SARM1 for a Toll/interleukin-1 receptor (TIR) domain is annotated in the C-terminal region 10-12. The properties and biological functions of SARM1 have been investigated in the immune and nervous systems in the past decades 13-16. Accumulating studies have shown that SARM1 plays multiple roles in defense of pathogen infection and brain development. On the one hand, human SARM1 negatively regulates MYD88- and TRIF-dependent TLRs signaling in immune responses 17, 18. On the other hand, SARM1 influences cell death and axonal degeneration, therefore, mediates the neurodegenerative processes of WD 19, 20. Using SARM1 knockout mice, it has been demonstrated that loss of SARM1 in neurons prevents cell death under oxygen and glucose deprivation stress 15. Loss of SARM1 effectively suppresses WD for weeks after axotomy, indicating that SARM1 plays a critical role in WD 16. In the CNS, lack of SARM1 in mice improves functional outcomes and attenuates traumatic axonal injury 21, however, loss of SARM1 does not suppress axonal degeneration in SOD1^G93A^ mouse model of amyotrophic lateral sclerosis (ALS) 22. Interestingly, recent studies have shown that SARM1 regulates neuronal intrinsic immune response to traumatic axonal injuries in the sciatic nerve injury model 23. These results suggest that SARM1 plays differential roles under different axonal injury models. However, the exact functions of SARM1 and the underlying mechanisms after SCI still remain poorly understood. Moreover, due to the diverse functions of SARM1, it is necessary to make SARM1 conditional knockout (CKO) mice to address the detailed functions of SARM1 under different pathological conditions.

In the present studies, we examined the roles of SARM1 after SCI. We found that SARM1 was upregulated in neurons and astrocytes of the spinal cords at early stage after SCI, and conditional deletion of SARM1 in neurons and astrocytes reduced neuroinflammation at SCI early phase through reduction of NF-κB signaling, which might be due to upregulation of HSP70, and promoted axonal regeneration at SCI intermediate phase. Moreover, similar results were obtained by FK866 (an inhibitor of SARM1) treatment for SCI, which may help to develop new strategies for the treatment of SCI.

## Materials and Methods

### Animals and mouse breeding

SARM1^Nestin^-CKO conditional knockout mice were generated by crossing the floxed SARM1 allele (SARM1^f/f^) mice with Nestin-Cre transgenic mice (003771, from The Jackson Laboratory; donated by Dr. Rudiger Klein), which expresses Cre recombinase in neural stem cells under the control of the Nestin promoter and conditional knockout genes in neural stem cells and as well as their derivatives including neurons and astrocytes 24. SARM1^GFAP^-CKO conditional knockout mice were generated by crossing SARM1^f/f^ mice with GFAP-Cre transgenic mice (004600, from The Jackson Laboratory; donated by Dr. Albee Messing) 25. SARM1^f/f^ mice were obtained by crossing between SARM1^f/w^ mice, which were generated by Shanghai Biomodel Organism Science & Technology Development Co., Ltd. A targeting vector containing the first two exons of the SARM1 gene was created by recombineering. Briefly, transformed ES colonies were screened by long-template PCR with the following primer sets: P5F (5'-GGAGTTATAGAGGATCACGAGCCAC-3') and P5R (5'-GGCCTACCCGCTTCCATTGCTC-3') to generate a 5.1-kb band for positive clones; P3F (5'-CCGTGCCTTCCTTGACCCTGG-3') and P3R (5'-AGCCTTTGCCCACTGAGACATC-3') to generate a 4.7-kb band for positive clones. Successfully targeted ES clones (confirmed by both 5'PCR and 3'PCR) were microinjected into C57BL/6J blastocysts. Germline transmission from generated chimeric offspring was confirmed by long-template PCR. Mice carrying the targeted allele were bred to Flp recombinase transgenic mice to remove the FRT-flanked Neo cassette and to generate the SARM1 flox mice. Genomic DNAs extracted from tail biopsies were genotyped with a PCR primer set (P1: 5'-AGCAACAAGCACTCTGAATGG-3', P2: 5'-AGATCACGCCTAGACCGATG-3') that generated a 466-bp band from the wild-type allele, a 500-bp band from the SARM1 floxed allele. Flp was isolated by crossing SARM1^f/w^; Flp mice with wild-type mice. Genomic DNAs extracted from tail biopsies were genotyped with a PCR primer set (PA: 5'-CACTGATATTGTAAGTAGTTTGC-3', PB: 5'-CTAGTGCGAAGTAGTGATCAGG-3') that generated no band from the wild-type allele, a 715-bp band from the Flp allele. Nestin-Cre-tdTomato reporter (Nestin-Cre^+/-^; Ai14) mice were generated by crossing the floxed tdTomato at Rosa 26 locus allele (Ai14) (gifted from Xiao-Ming Li's Lab, Zhejiang University) with Nestin-Cre transgenic mice. All wild-type, SARM1^f/w^, SARM1^f/f^, Nestin-Cre^+/-^, GFAP-Cre^+/-^, Nestin-Cre^+/-^; SARM1^f/w^, GFAP-Cre^+/-^; SARM1^f/w^, SARM1^Nestin^-CKO, SARM1^GFAP^-CKO and Nestin-Cre^+/-^; Ai14 mice were maintained in C57BL/6J strain background. For all experiments, 8-12 weeks old male mice were used unless specifically stated. In all studies, at least three pairs of mice from same litters were used. Significant efforts were also made to minimize the total number of animals used while maintaining statistically valid group numbers. All experimental animals were approved by the Laboratory Animals Ethics Committee of Wenzhou Medical University.

### SCI surgical procedures

All of the animals (2 M male wild-type, SARM1^f/f^, SARM1^Nestin^-CKO mice and SARM1^GFAP^-CKO mice) underwent general anesthesia (20 ml/kg) by intraperitoneal injection of avertin (2, 2, 2-tribromoethanol, Sigma-Aldrich) in 0.9% saline solution. Surgical procedures were described previously 26, 27. Briefly, a laminectomy from T8 to T10 of spinal cords was performed on a surgical microscope (Nikon SMZ745) and a mouse spinal cord adapter (RWD, 68094) was used, and the bilateral pedicles were firmly fixed by two toothed retaining rods to make sure that there was no vertebral movement in our SCI model. Spinal cord was contused in T9 by a weight (10 g) from 5 cm height on a mouse spinal cord impactor (RWD, 68097), which caused a very grave injury, but was commonly used for SCI in mice in several previous studies 26, 27. The legs of the mice were stretched and turned, and the drooped tails indicated the success of the contusion SCI model. After disinfected with povidone-iodine solution and washed with saline, the muscles were sutured layer by layer and the skin was stapled. The mice were rehydrated with 2 ml of 0.9% NaCl (subcutaneous injection) and kept warm in an incubator. After waking up, they were transferred to clean cages in a warm room with easily accessible feed and water. Their water intake, temperature, and body weight were checked every day until they recovered an ascending weight curve 28. After SCI, the bladder was manually evacuated twice daily until the restoration urinating function 29. In sham group, all animals were subjected to laminectomy alone. All animals were randomly distributed into the following groups: the sham group, the “1 d after SCI” group, the “3 d after SCI” group, the “7 d after SCI” group, the “14 d after SCI” group and the “28 d after SCI” group and evaluated blind to genotype and experimental condition. Mice were utilized to assess histological, biochemical and behavioral function procedures as described below.

### Behavioral analysis

Mice were evaluated using four behavioral experiment assays to assess hindlimb functions as previously described 30.

### Footprint analysis

To assess the athletic ability of forelimbs and hindlimbs, mice were running along a paper-lined runway, as described before 31, 32. Each forelimb and hindlimb was brushed with black (forelimbs) and red (hindlimbs) nontoxic ink, and qualitative analyzed plantar stepping, stride length and width, and overall stepping ability.

### Rotarod performance

To evaluate the function of balance, grip strength and motor coordination, animals were put in a single-lane rotarod (Anhui Zhenghua Biological Instrument Equipment, YLS-10A) for three trials per session, which was set for 3 to 30 rpm over 180 sec, and scored on seconds to fall.

### Pole test

To evaluate the ability of balance and coordination, animals were placed on a 50 cm-high pole and the time all four limbs land on were recorded. When the animal paused and could not turn but instead descended with a lateral body position, the trial was repeated. Each trial was scored individually and averaged for a final score per session.

### Open-field locomotor task

The objective of this evaluation was to assess gross voluntary use of the hindlimbs, and did not attempt to define subtle differences in usage that might be correlated with specific neural mechanisms that might underlie dysfunctions. A simple six-point scale was used, as described before 32, 33. All animals were evaluated in an open field by the same one or two observers blind to the experimental condition and received a score for gross voluntary movement of each hindlimb using an operationally defined six-point scale: (0) no voluntary hindlimb movement, (1) little voluntary hindlimb movement, (2) hindlimb movements obvious but did not assist in weight support or stepping, (3) hindlimb assisted in occasional weight support and plantar placement but not in stepping, (4) hindlimb used for weight support and stepping, but obvious disability, and (5) hindlimb function essentially normal.

### Basso Mouse Scale (BMS) scoring analysis

BMS scoring analysis was conducted to detect variations in locomotive function of hind limbs in mice after SCI. All animals were evaluated by the same one or two observers blind to the experimental condition and were scored from 0 to 9 points based on the scoring system (posterior ankle joint mobility, coordination, paw posture, trunk stability, and tail posture) as previously described 34, 35.

### Western blot

Spinal cords and other nerve tissues were lysed in the lysis buffer: ice-cold RIPA Buffer (P0013B, Beyotime), 100 mM NaF, 100 mM Na_3_VO_4_, 100 mM PMSF (ST506, Beyotime), and incubated at 4 °C for 30 min, and centrifuged at 12,000 rpm for 30 min, and extracted with 5 × loading buffer (P1040, Solarbio). Finally, the lysates were boiled at 100 °C for 10 min. The samples were separated using 8%, 10% or 12% sodium dodecyl sulfate-polyacrylamide gel electrophoresis (SDS-PAGE) and transferred onto nitrocellulose membranes (Life sciences, USA). After blocking in 5% skim milk (#232100, BD Bioscience) for 1.5 h, the immunoblots were incubated with different primary antibodies for overnight at 4 °C. Primary antibodies included mouse anti-β-actin (A5316, Sigma-Aldrich, 1:10,000), rabbit anti-SARM1 (ab226930, Abcam, 1:1,000), rabbit anti-NF-κB (p65) (ab16502, Abcam, 1:1,000), rabbit anti-IKB-α (ab32518, Abcam, 1:1,000), rabbit anti-p-JNK (#4668, Cell Signaling, 1:1,000), rabbit anti-JNK (bs-2592R, Bioss, 1:1,000), rabbit anti-c-Jun (bs-0670R, Bioss, 1:1,000), rabbit anti-Hspa1a (A0284, ABclonal, 1:1,000). After washed for three times, the blots were then incubated in room temperature for 1.5 h with the secondary antibodies, goat anti-mouse IgG-HRP (#31460, Pierce, 1:5,000) or goat anti-rabbit IgG-HRP (#31420, Pierce, 1:5,000). The western blots were detected by the ECL detection kit (Bio-Rad, USA). Subsequently, blots were analyzed using Quantity One software (Bio-Rad, USA).

### Nissl staining

Nissl staining was performed as previously described 36. After mice were perfused with 0.1 mol/L PBS followed by 4% paraformaldehyde (PFA), the spinal cords were immersed in 4% PFA for 24 h and transferred to 30% sucrose solution until they sank. Subsequently, the spinal cords were cut into 20-um-thick transverse and horizontal sections using a freezing microtome (Thermo, USA). After the sections were incubated with 0.1% cresyl violet for 5 min at room temperature, the sections were rinsed in double distilled water followed by 95% ethanol, dehydrated in 100% ethanol and cleared in xylene, and covered by neutral resins. The images were acquired with a microscope (Nikon, Tokyo, Japan) and the ventral horn neurons were counted with Image J software (Media Cybernetics, Bethesda, MD, USA). Quantitative analysis of histological staining and fluorescence was used by Image J.

### Hematoxylin-Eosin (HE) staining

Briefly, after perfusion with 0.1 M PBS followed by 4% PFA, the spinal cords of mice were immersed in 4% PFA for 24 h and transferred to 30% sucrose solution until they sank. Subsequently, the spinal cords were embedded in OCT (optimal cutting temperature) and were cut into 20 μm-thick transverse sections using a freezing microtome (Thermo, USA). After staining with hematoxylin for 1 min, the sections were washed three times in double distilled water. Then the sections were incubated in the acidic liquid alcohol differentiation for 30 s, stained with eosine for 50 s, followed by 95% ethanol, 100% ethanol, and finally cleared in xylene, and mounted by neutral resins. The images were acquired with a microscope (Nikon, Tokyo, Japan) and quantitative analysis of the images was done by Image J.

### Immunostaining

For staining of the spinal cords tissue sections, after fixed 30 min and antigen repaired 30 min at 90 °C by sodium citrate antigen retrieval solution (C1032, Solarbio), the spinal cords tissue sections were processed for immunostaining by 1 h blocking in 5% BSA (4240GR100, Biofroxx) plus 0.3% Triton X-100 (T8200, Solarbio) at room temperature, for overnight incubation with primary antibodies at 4 °C, and washed three times in PBS and then were incubated for 1 h at room temperature with appropriate secondary antibodies. Primary antibodies included mouse anti-NeuN (ab177487, Abcam, 1:500), mouse anti-Aldh1l1 (ab56777, Abcam, 1:500), mouse anti-GFAP (MAB360, Millipore, 1:500), goat anti-Iba1 (ab5076, Abcam, 1:500), mouse anti-MBP (ab62631, Abcam, 1:500), rabbit anti-SARM1 (ab226930, Abcam, 1:500), rabbit anti-CD45 (ab10558, Abcam, 1:500), rabbit anti-NF (ab8135, Abcam, 1:500), rabbit anti-GAP43 (ab16053, Abcam, 1:500). Secondary antibodies included donkey anti-rabbit Alexa Fluor488 (A21206, Invitrogen, 1:1,000), donkey anti-mouse Alexa Fluor488 (A21202, Invitrogen, 1:1,000), donkey anti-rabbit Alexa Fluor546 (A10040, Invitrogen, 1:1,000), donkey anti-mouse Alexa Fluor546 (A10036, Invitrogen, 1:1,000), donkey anti-goat Alexa Fluor488 (A11055, Invitrogen, 1:1,000). Images were acquired using confocal microscopes (TCS SP8, Lecia) or microscope (Li2, Nikon) and analyzed with Image J and Photoshop (Adobe).

### Quantitative Real-Time PCR (qRT-PCR)

For qRT-PCR, total RNA was extracted from spinal cords of SARM1^f/f^ or SARM1^Nestin^-CKO mice at 3 d after SCI using TRIzol^TM^ reagent (#15596026, Ambion) according to the protocol provided by the manufacturer. Then, RNA was reversely transcribed into cDNA with a SuperScript^TM^ One-Step Reverse Transcription Kit (#10928-034, Invitrogen, CA, USA). The expression levels of mRNA were quantified using the iTaq^TM^ Universal SYBR Green Supermix (Bio-Rad, USA) on the Real-Time PCR detection System (Applied Biosystems, USA). Samples were amplified independently at least three times. Relative gene expression was converted using the 2^-ΔΔCt^ method against β-actin. β-actin primer: forward, 5'-GTGACGTTGACATCCGTAAAGA-3' and reverse, 5'-GCCGGACTCATCGTACTCC-3' 37. NF-κB primer: forward, 5'-AGAGGGGATTTCGATTCCGC-3' and reverse, 5'-CCTGTGGGTAGGATTTCTTGTTC-3' 38. IFN-α primer: forward, 5'-GGATGTGACCTTCCTCAGACTC-3' and reverse, 5'-ACCTTCTCCTGCGGGAATCCAA-3' 39. IFN-β primer: forward, 5'-GCCTTTGCCATCCAAGAGATGC-3' and reverse, 5'-ACACTGTCTGCTGGTGGAGTTC-3' 40. IFN-γ primer: forward, 5'-AGCGGCTGACTGAACTCAGATTGTAG-3' and reverse, 5'-GTCACAGTTTTCAGCTGTATAGGG-3' 41. IL-1β primer: forward, 5'-TGGACCTTCCAGGATGAGGACA-3' and reverse, 5'-GTTCATCTCGGAGCCTGTAGTG-3' 42. MIP-1α (CCL3) primer: forward, 5'-ACTGCCTGCTGCTTCTCCTACA-3' and reverse, 5'-ATGACACCTGGCTGGGAGCAAA-3' 43. TNF-α primer: forward, 5'-GGTGCCTATGTCTCAGCCTCTT-3' and reverse, 5'-GCCATAGAACTGATGAGAGGGAG-3' 44. RANTES primer: forward, 5'-CTCACCATATGGCTCGGACA-3' and reverse, 5'-ACAAACACGACTGCAAGATTGG-3' 45.

### RNA sequencing and functional enrichment analysis

RNA sequencing was performed by the Novogene Bioinformatics Institute (Beijing, China). The mRNA of spinal cords of SARM1^f/f^ and SARM1^Nestin^-CKO mice were collected. After perfusion with 0.1 M PBS, the tissues were frozen with liquid nitrogen immediately. The extraction of total RNAs was performed using the RNeasy Mini kit (Qiagen) according to the manufacturer's protocol. RNA purity was assessed using the ND-1000 Nanodrop. The ratio of A260 to A280 and A260 to A230 for each RNA sample was above 1.8 and 2.0, respectively. RNA integrity was measured using the RNA Nano 6000 Assay kit of the Bioanalyzer 2100 system (Agilent Technologies, CA, USA). Sequencing libraries were accomplished using NEBNext® UltraTM RNA Library Prep Kit for Illumina® (NEB, USA) following manufacturer's recommendations and index codes were added to attribute sequences to each sample. After that, the library fragments were purified using AMPure XP system (Beckman Coulter, Beverly, USA) and then the cDNA fragments of 250~300 bp were selected. Fragments were then amplified by 10 cycles of PCR using Phusion High-Fidelity DNA polymerase and library quality was assessed on the Agilent Bioanalyzer 2100 system. Following cluster generation, the library preparations were then sequenced on an Illumina Novaseq platform, by that, 150 bp paired-end reads were generated. And then the mapped reads were assembled by StringTie (v1.3.3b) and the number of fragments per kilobase of transcript sequence per million base pairs sequenced (FPKM) was obtained. Reference genome and gene model annotation were gained from genome website. Gene set enrichment analysis was used for enrichment analysis. The statistical significance of signature enrichment was assessed using 1000-gene-set permutations.

### Pharmacological interference with FK866 or apoptozole

Sham control and spinal cords injured C57BL/6J mice were treated with FK866 (10 mg/kg i.p., Sigma-Aldrich, catalog #F8557) twice every day until the experiments were terminated 46, 47. Meanwhile, the control group mice were injected intraperitoneally with the same amount of normal saline. For apoptozole (MedChemExpress, HY-15098) treatment, SARM1^Nestin^-CKO mice were injected intraperitoneally at a dose of 4 mg/kg immediately after SCI and every other day until the experiments were terminated 48.

### Statistical analysis

All data presented represent results as mean ± SEM from at least three independent experiments. Statistical analysis was performed using Student's *t*-test or using ANOVA with Bonferroni post-tests. Statistical significance was defined as *P* < 0.05.

## Results

### SARM1 was upregulated in neurons and astrocytes at early stage after SCI

**Figure 1:**
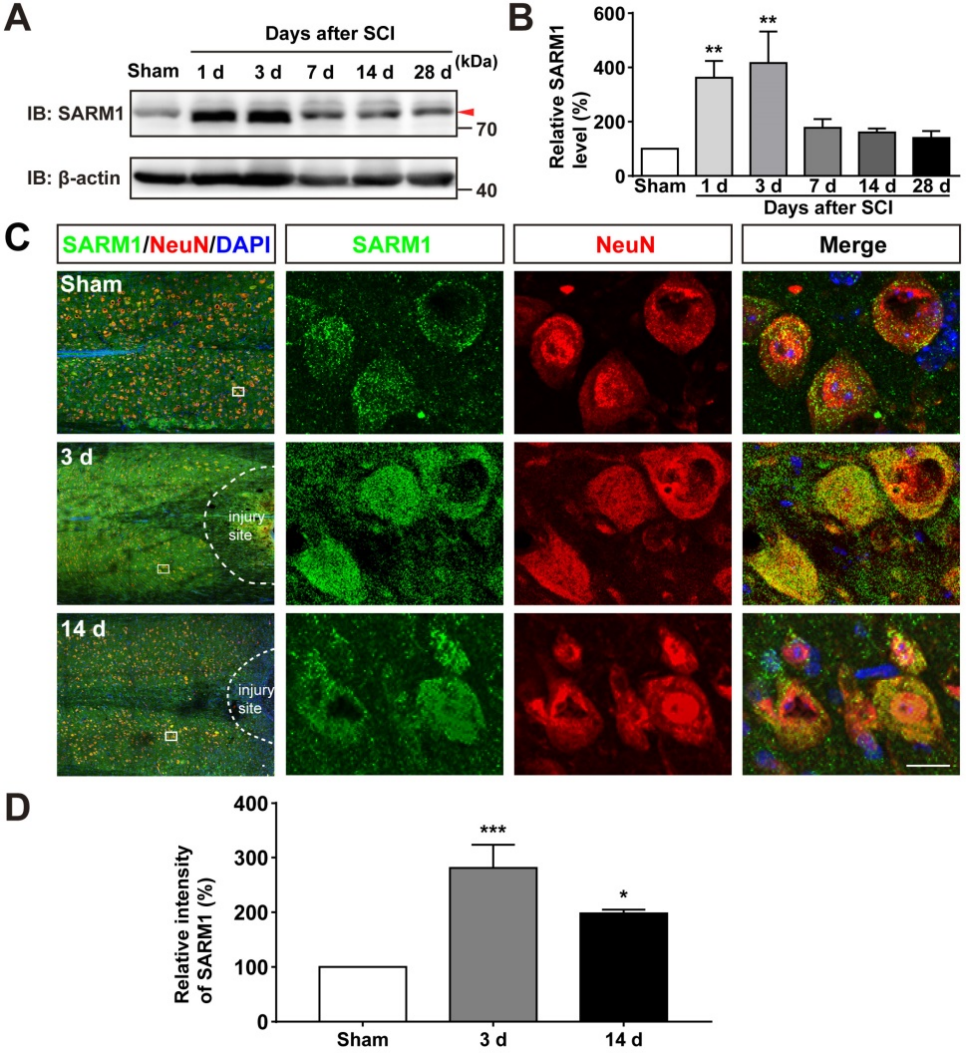
**SARM1 was upregulated in neurons at early stage after SCI. (A)** Western blot analysis of SARM1 expression in the spinal cords at different stages after SCI. **(B)** Quantitative analysis of the relative SARM1 level (normalized to sham group) as shown in (A) (*n* = 5 per group). **(C)** Double immunostaining analysis of SARM1 (green) and NeuN (red) in coronal sections of uninjured spinal cords and injured spinal cords at 3 d and 14 d after SCI. **(D)** Quantitative analysis of the relative fluorescent intensity of SARM1 level (normalized to sham group) at different stages after SCI as shown in (C) (*n* = 5 per group). Dashed lines indicated the outline of the injury sites. Images of selected regions (rectangles) in (C) were shown at higher magnification. Scale bars, 20 µm. Data were mean ± SEM. One-way ANOVA with Bonferroni's post-tests, ^*^P < 0.05, ^**^P < 0.01, ^***^P < 0.001.

To investigate the function of SARM1 in spinal cords, we first examined the expression pattern of SARM1 in the spinal cords, and brain regions such as cortex, hippocampus, cerebellum, olfactory bulb and midbrain by Western blot. As shown in Figure S1A-B, we found SARM1 was expressed in the spinal cords, but lower than other brain regions. To further know the spatial distribution of SARM1 in the spinal cords, double immunostaining of SARM1 and several cell markers including NeuN (a marker of neurons), GFAP, Aldh1l1 (markers of astrocytes) and Iba1 (a marker of microglia) were performed. We found that SARM1 was mainly detected in NeuN^+^ neurons, weakly in GFAP^+^ and Aldh1l1^+^ astrocytes, but not in Iba1^+^ microglial cells (Figure S1C-F). To explore the potential functions of SARM1 after SCI, contusion SCI model was established. As shown in Figure S2, the moderate-severe contused injury of spinal cord in T9 was made by a weight (10 g) from 5 cm height that caused a clear injury site and motor impairments. Based on this SCI model, the expression pattern of SARM1 in the spinal cords was detected at different stages after SCI by western blot. We found that the expression of SARM1 protein was significantly upregulated at the 1 d, 3 d at the injury site, and with a peak between 3 d and 7 d after SCI (Figure 1A-B). Furthermore, double immunostaining showed that SARM1 protein was upregulated and displayed the cytoplasmic location in the NeuN^+^ neurons (Figure 1C-D) and GFAP^+^ astrocytes (Figure S3A-C), but not in Iba1^+^ microglia (Figure S3D), at 3 d after SCI and reached the base level at 14 d after SCI. Taken together, these results indicated that SARM1 was mainly upregulated in neurons and astrocytes at early stages after SCI, which might be involved in the neuronal regeneration.

### Normal development of spinal cords and motor function in SARM1^Nestin^-CKO mice

To further study the functions of SARM1 in SCI, conditional knockout SARM1 in neurons and astrocytes mice, SARM1^Nestin^-CKO mice, were generated by crossing SARM1^f/f^ mice with Nestin-Cre transgenic mice (Figure S4A-C). In order to verify the specific tissue cells of the Nestin-Cre expression, Nestin-Cre-tdTomato reporter (Nestin-Cre^+/-^; Ai14) mice were also generated. Immunostaining showed that tdTomato co-stained with NeuN^+^ neurons and GFAP^+^ astrocytes in the Nestin-Cre-tdTomato reporter mice (Figure S4D-G), suggesting that Nestin-Cre mice were suitable to delete genes in neurons and astrocytes. Indeed, as shown in Figure S4H-I, the expression of SARM1 was dramatically decreased in the spinal cords and other brain regions such as cortex, hippocampus and cerebellum of SARM1^Nestin^-CKO mice, compared with control mice.

**Figure 2:**
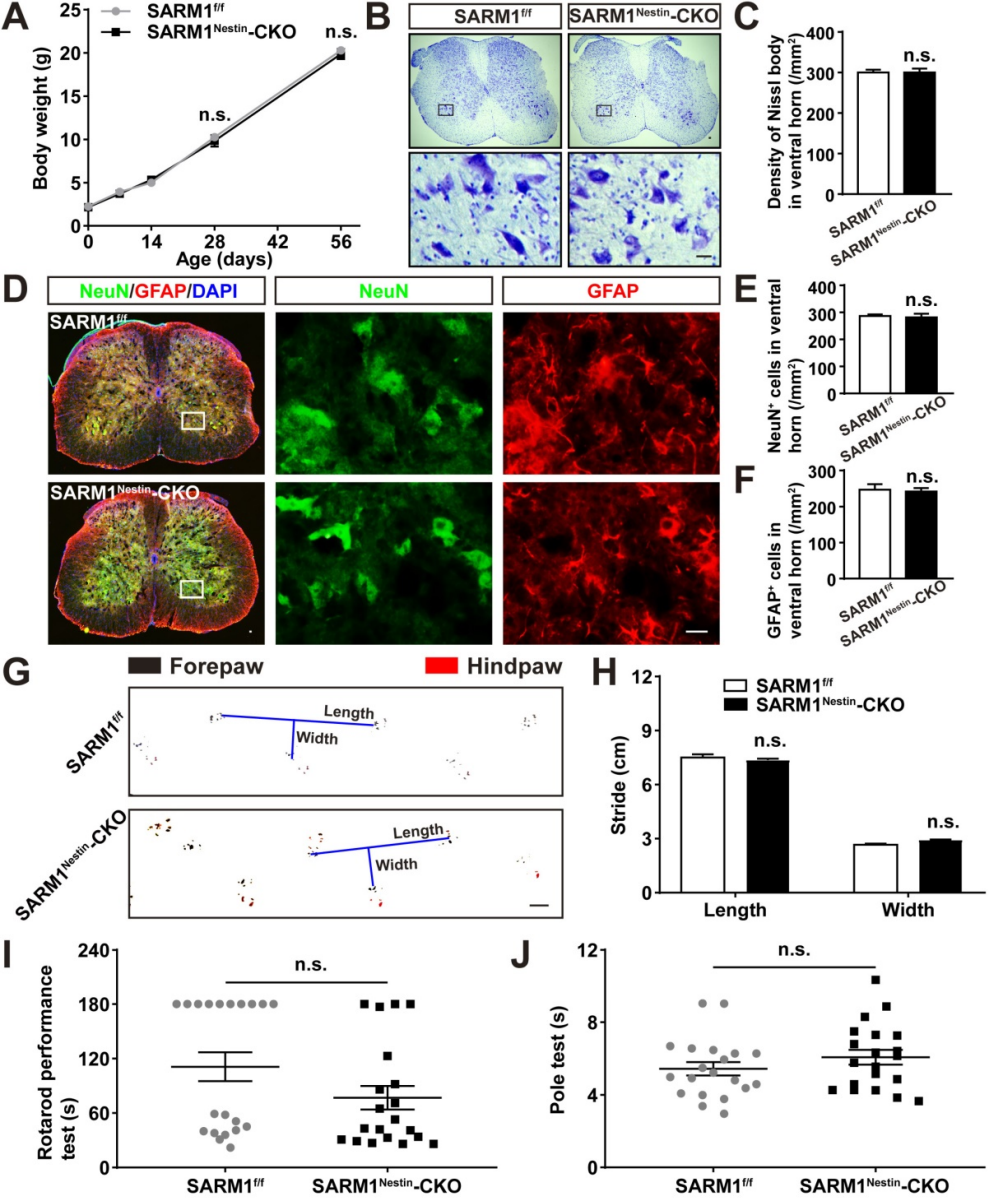
**Normal development of spinal cords and motor function of SARM1^Nestin^-CKO mice. (A)** Quantitative analysis of the body weight of SARM1^f/f^ and SARM1^Nestin^-CKO mice at different developmental stages (two-way ANOVA (repeated measures) with Bonferroni's post-tests, *n* = 3 per group). **(B)** Nissl staining images showing the nissl bodies in the ventral horn of the spinal cords of 2 M male SARM1^f/f^ and SARM1^Nestin^-CKO mice. **(C)** Quantitative analysis of the number of neurons as shown in (B) (*n* = 6 per group). **(D)** Double immunostaining analysis of NeuN (green) and GFAP (red) in the ventral horn of the spinal cords of 2 M male SARM1^f/f^ and SARM1^Nestin^-CKO mice.** (E-F)** Quantitative analysis of the number of NeuN^+^ or GFAP^+^ cells as shown in (D) (*n* = 6 per group). **(G)** Representative footprint images of 2 M male SARM1^f/f^ and SARM1^Nestin^-CKO mice. **(H)** Quantitative analysis of stride length and stride width in footprint assays of 2 M male SARM1^f/f^ and SARM1^Nestin^-CKO mice as shown in (G) (*n* = 6 per group). **(I)** Quantitative analysis of the time taken to fall in rotarod performance test of 2 M male SARM1^f/f^ and SARM1^Nestin^-CKO mice (*n* = 20 per group)**. (J)** Quantitative analysis of the time all the four limbs took to land on in the pole test of 2 M male SARM1^f/f^ and SARM1^Nestin^-CKO mice (*n* = 20 per group). Images of selected regions (rectangles) in (B) and (D) were shown at higher magnification. Scale bars, 20 µm (B, D), 1cm (G). Data were mean ± SEM. Two-tailed Student's *t*-test, n.s. not significant (*P* > 0.05).

We next examined whether the conditional deletion of SARM1 in neurons and astrocytes affected the spinal cords development and motor functions. As shown in Figure 2A-F, there was no significant difference in the body weight, neuronal number and neuronal distribution in the spinal cords between SARM1^f/f^ and SARM1^Nestin^-CKO mice. Moreover, we found that conditional deletion of SARM1 in neurons and astrocytes did not affect the motor function of mice based on the behavior tests including footprint, rotarod performance and pole test assays (Figure 2G-J). Taken together, these results indicated that conditional deletion of SARM1 in neurons and astrocytes did not affect the development of spinal cords and motor functions.

### Conditional deletion of SARM1 in neurons and astrocytes promoted the functional recovery of behavior performance after SCI

**Figure 3:**
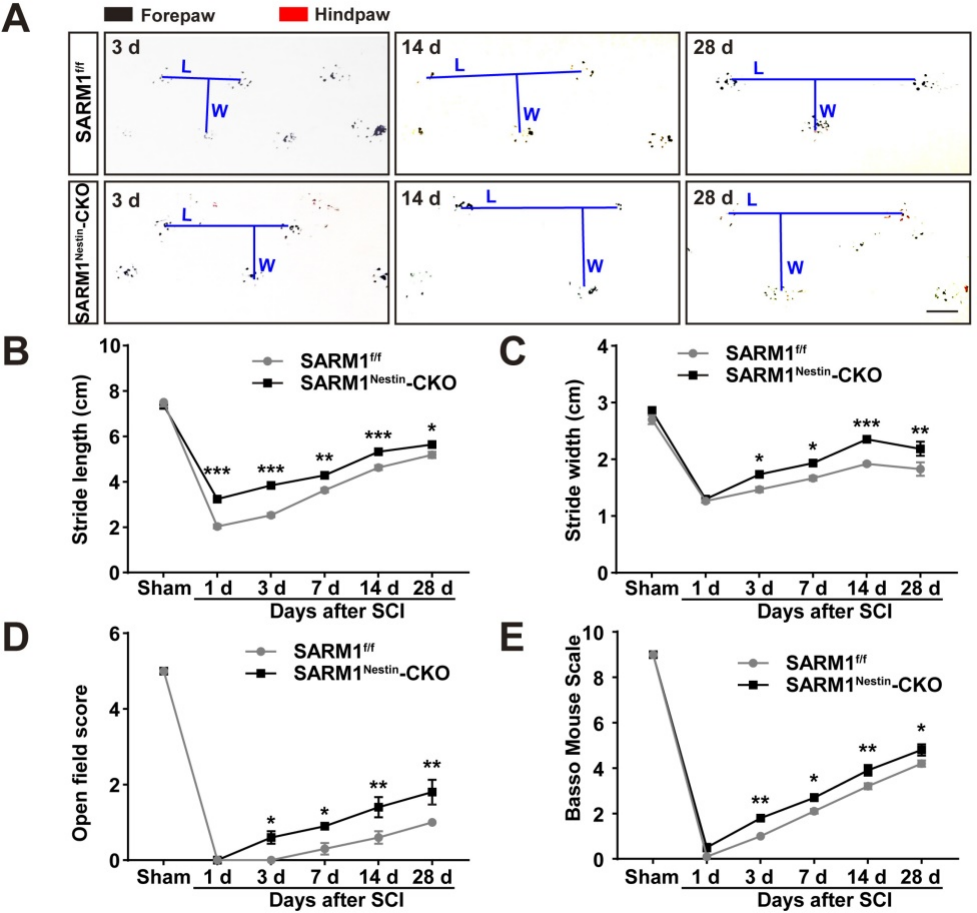
** Conditional deletion of SARM1 in neurons and astrocytes promoted the recovery of behavior performance after SCI. (A)** Representative footprint images of SARM1^f/f^ and SARM1^Nestin^-CKO mice at 3 d, 14 d and 28 d after SCI. **(B-C)** Quantitative footprint analysis of stride length (B) and stride width (C) in footprint behavioral assay at different stages of SARM1^f/f^ and SARM1^Nestin^-CKO mice after SCI (*n* = 6 per group). **(D-E)** Quantitative analysis of gross voluntary movement in open-field walking (D, *n* = 10 per group) and BMS scoring analysis (E, *n* = 10 per group) of SARM1^f/f^ and SARM1^Nestin^-CKO mice over a 28-d period after SCI. Scale bars, 1 cm. Data were mean ± SEM. Two-way ANOVA (repeated measures) with Bonferroni's post-tests, ^*^P < 0.05, ^**^P < 0.01, ^***^P < 0.001.

The upregulation of SARM1 mainly in neurons and astrocytes led to the speculation for a critical role of SARM1 in neuronal regeneration after SCI. We first examined whether the motor function was affected by SARM1 deletion in neurons and astrocytes after SCI. As shown in Figure 3A-C, the footprint behavioral assays showed better performance of SARM1^Nestin^-CKO mice in stride length at 1 d, 3 d, 7d, 14 d, 28 d and stride width at 3 d, 7 d, 14 d, 28 d after SCI. The gross voluntary movement in the open-field locomotor task and BMS scoring analysis of SARM1^Nestin^-CKO mice were significantly better than SARM1^f/f^ mice at 3 d, 7 d, 14 d, 28 d, but was not comparable with SARM1^f/f^ mice at 1 d after SCI (Figure 3D-E).

**Figure 4:**
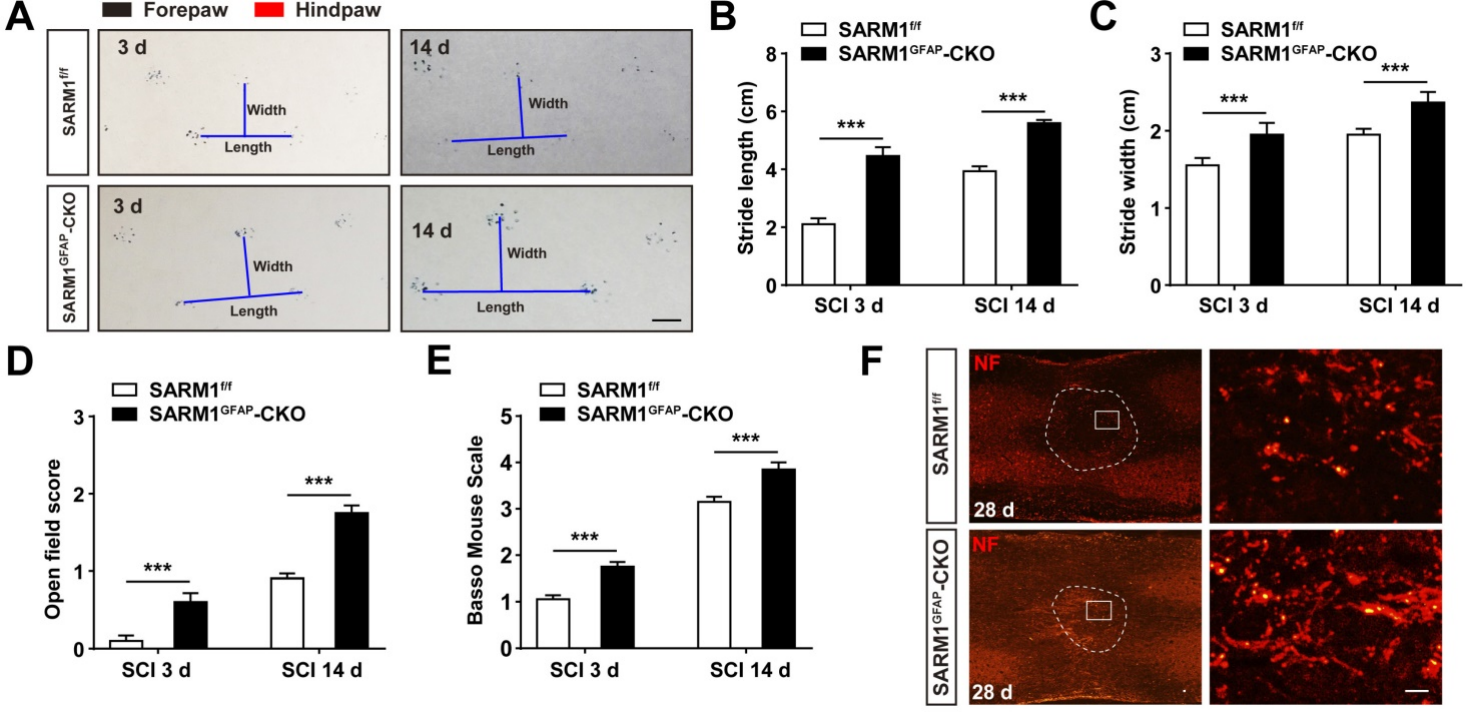
** Conditional knockout SARM1 in astrocytes also improved the functional recovery of mice after SCI. (A)** Representative footprint images of 2 M male SARM1^f/f^ and SARM1^GFAP^-CKO mice at 3 d and 14 d after SCI. **(B-C)** Quantitative analysis of stride length (B) and stride width (C) in footprint assays of 2 M male SARM1^f/f^ and SARM1^GFAP^-CKO mice as shown in (A) (*n* = 6 per group). **(D)** Quantitative analysis of gross voluntary movement in open-field test of 2 M male SARM1^f/f^ and SARM1^GFAP^-CKO mice at 3 d and 14 d after SCI (*n* = 20 per group).** (E)** Quantitative analysis of BMS scoring of 2 M male SARM1^f/f^ and SARM1^GFAP^-CKO mice at 3 d and 14 d after SCI (*n* = 20 per group). **(F)** Immunostaining analysis of NF (red) in the spinal cords of SARM1^f/f^ and SARM1^GFAP^-CKO mice at 28 d after SCI. Dashed lines indicated the outline of the injury sites. Images of selected regions (rectangles) in (F) were shown at higher magnification. Scale bars, 1cm (A), 20 µm (E). Data were mean ± SEM. Two-tailed Student's *t*-test, ^***^P < 0.001.

To further address the role of SARM1 in astrocytes after SCI, SARM1^GFAP^-CKO mice were generated by SARM1^f/f^ mice with GFAP-Cre transgenic mice (Figure S5A-B). Western blot showed that SARM1 was significantly decreased in the spinal cords and other brain regions such as cortex, hippocampus and cerebellum of SARM1^GFAP^-CKO mice (Figure S5C-D). As shown in Figure S5E-H, conditional knockout SARM1 in astrocytes did not affect the normal development and motor function based on the behavior tests including footprint, rotarod performance and pole test assays. However, after SCI, the footprint behavioral assays showed better performance of SARM1^GFAP^-CKO mice in stride length and width at 3 d and 14 d (Figure 4A-C). The gross voluntary movement in the open-field locomotor task and BMS scoring analysis of SARM1^GFAP^-CKO mice were significantly better than SARM1^f/f^ mice (Figure 4D-E).

Taken together, these results suggested that conditional deletion of SARM1 in neurons and astrocytes promoted the recovery of motor functions in some behavior tests after SCI.

### Conditional deletion of SARM1 in neurons and astrocytes inhibited the loss of neurons and the neuronal degeneration at intermediate phase after SCI

**Figure 5:**
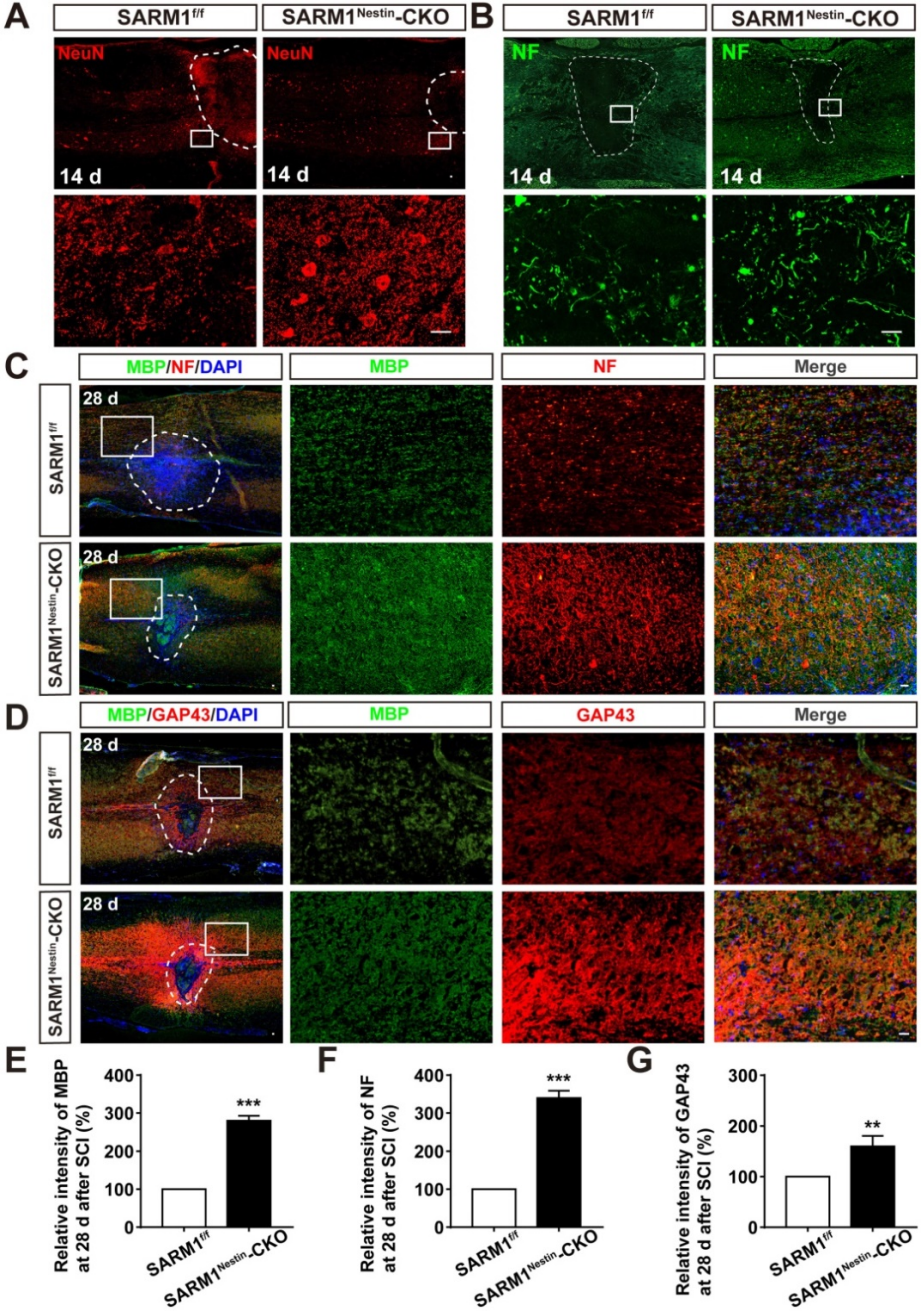
** Conditional deletion of SARM1 in neurons and astrocytes promoted the neuronal regeneration at intermediate phase after SCI. (A)** Immunostaining analysis of NeuN (red) in the spinal cords of SARM1^f/f^ and SARM1^Nestin^-CKO mice at 14 d after SCI. **(B)** Immunostaining analysis of NF (green) in the spinal cords of SARM1^f/f^ and SARM1^Nestin^-CKO mice at 14 d after SCI. **(C)** Double immunostaining analysis of MBP (green) and NF (red) in the spinal cords of SARM1^f/f^ and SARM1^Nestin^-CKO mice at 28 d after SCI. **(D)** Double immunostaining analysis of MBP (green) and GAP43 (red) in the spinal cords of SARM1^f/f^ and SARM1^Nestin^-CKO mice at 28 d after SCI. **(E)** Quantitative analysis of the intensity of MBP as shown in (C) (*n* = 6 per group, normalized to SARM1^f/f^ mice group). **(F)** Quantitative analysis of the intensity of NF as shown in (C) (*n* = 6 per group, normalized to SARM1^f/f^ mice group). **(G)** Quantitative analysis of the intensity of GAP43 as shown in (D) (*n* = 3 per group, normalized to SARM1^f/f^ mice group). Dashed lines indicated the outline of the injury sites. Images of selected regions (rectangles) in (A-D) were shown at higher magnification. Scale bars, 20 µm. Data were mean ± SEM. Two-tailed Student's *t*-test, ^**^P < 0.01, ^***^P < 0.001.

Since SARM1 promotes the Wallerian Degeneration after injury as described previously 49-51, we next examined whether the deletion of SARM1 promoted the recovery of motor functions due to the delay in neuronal degeneration after SCI. The hematoma area in the spinal cords were comparable between SARM1^f/f^ mice and SARM1^Nestin^-CKO mice at 0 d after SCI (Figure S6), however, as expected, NeuN staining showed that the area of injury site and loss of neurons after SCI were significantly decreased in SARM1^Nestin^-CKO mice at 14 d after SCI, compared with SARM1^f/f^ mice (Figure 5A). These results suggested that SARM1 deletion alleviated the damage of neurons after SCI. Furthermore, we performed the immunostaining of neurofilament (NF) (an axonal regeneration marker) to assess the neural regeneration at 14 d after SCI, we found that the intensity of NF was significantly increased in SARM1^Nestin^-CKO mice, compared with the SARM1^f/f^ mice (Figure 5B). As shown in Figure 5C-D, and 5F-G, we found that the intensity of NF and GAP43 (another axonal regeneration marker) were significantly higher in SARM1^Nestin^-CKO mice at 28 d after SCI, compared with the SARM1^f/f^ mice. Meanwhile, we also found that the intensity of NF was significantly increased in SARM^GFAP^-CKO mice at 28 d after SCI, compared with the SARM1^f/f^ mice (Figure 4F). The intensity of myelin basic protein (MBP) (a mature myelin marker) was also significantly higher at 28 d after SCI in SARM1^Nestin^-CKO mice than SARM1^f/f^ mice (Figure 5C-E). Taken together, these results suggested that conditional deletion of SARM1 in neurons and astrocytes inhibited the loss of neurons and axonal degeneration at intermediate phase (2 w-6 M) after SCI.

### Conditional deletion of SARM1 in neurons and astrocytes reduced neuroinflammation at SCI early phase

**Figure 6:**
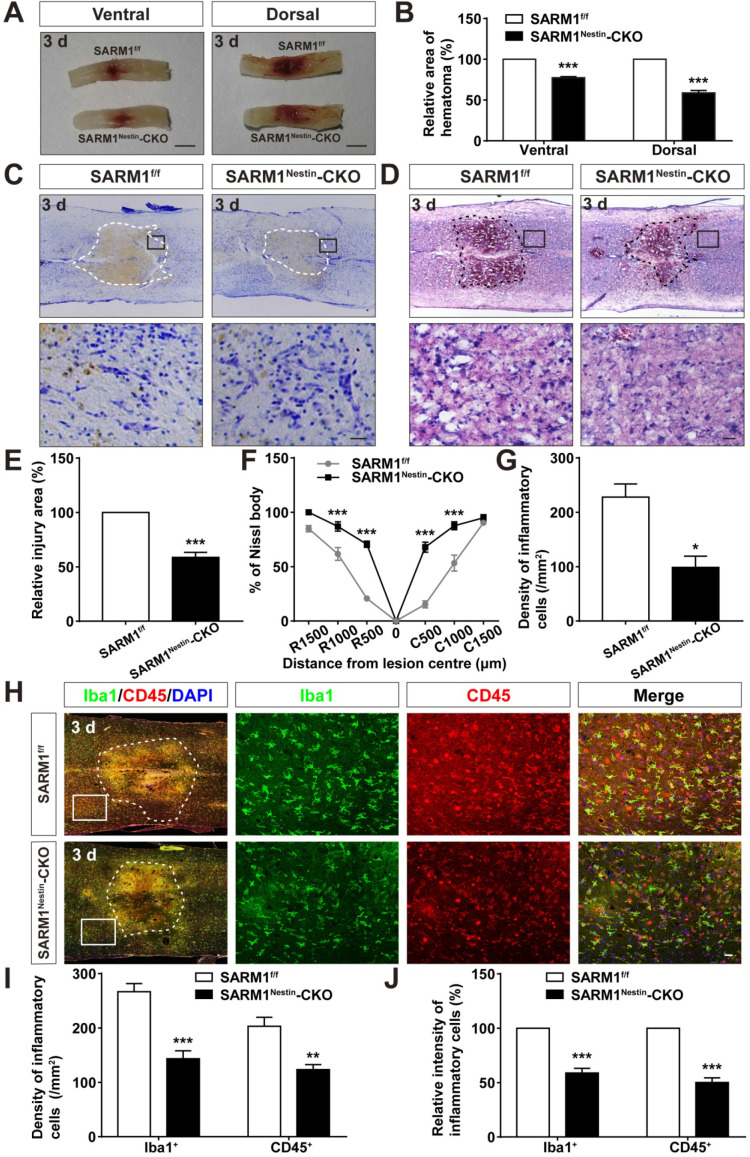
** Conditional deletion of SARM1 in neurons and astrocytes reduced the neuroinflammation at SCI early phase. (A)** Representative images of the ventral and dorsal spinal cords with hematoma of SARM1^f/f^ and SARM1^Nestin^-CKO mice at 3 d after SCI. **(B)** Quantitative analysis of hematoma area of the spinal cords as shown in (A) (*n* = 6 per group, normalized to SARM1^f/f^ mice group). **(C)** Nissl staining images showing the injury area in the spinal cords of SARM1^f/f^ and SARM1^Nestin^-CKO mice at 3 d after SCI. **(D)** HE staining images showing the inflammatory infiltration of the spinal cords of SARM1^f/f^ and SARM1^Nestin^-CKO mice at 3 d after SCI. **(E)** Quantitative analysis of the injury area in the spinal cords as shown in (C) (*n* = 3 per group, normalized to SARM1^f/f^ mice group). **(F)** Quantitative analysis of neurons by Nissl staining at various distances from the SCI lesion center as shown in (C) (two-way ANOVA (repeated measures) with Bonferroni's post-tests, *n* = 3 per group, normalized to SARM1^f/f^ mice group). **(G)** Quantitative analysis of the density of inflammatory cells in the spinal cords as shown in (D) (*n* = 3 per group).** (H)** Double immunostaining analysis of Iba1 (green) and CD45 (red) in the spinal cords of SARM1^f/f^ and SARM1^Nestin^-CKO mice at 3 d after SCI. **(I)** Quantitative analysis of the density of Iba1^+^ cells and CD45^+^ cells as shown in (H) (*n* = 6 per group).** (J)** Quantitative analysis of the intensity of Iba1^+^ cells and CD45^+^ cells as shown in (H) (*n* = 6 per group, normalized to SARM1^f/f^ mice group). Dashed lines indicated the outline of the injury sites. Images of selected regions (rectangles) in (C), (D), and (H) were shown at higher magnification. Scale bars, 3 mm (A), 20 µm (C, D, H). Data were mean ± SEM. Two-tailed Student's *t*-test, ^*^P < 0.05, ^**^P < 0.01, ^***^P < 0.001.

How does SARM1 deletion in neurons inhibit the neural degeneration after SCI? Since previous studies have shown that SARM1 regulates neuronal intrinsic immune response to axonal injury 23, we next examined whether SARM1 was involved in neuroinflammation after SCI. The spinal cords of SARM1^f/f^ and SARM1^Nestin^-CKO mice at 3 d after SCI were collected, as 3 d after SCI was at the early phase and SARM1 exhibited the highest expression as shown above. As shown in Figure 6A-B, area of hematoma was significantly decreased in SARM1^Nestin^-CKO mice at 3 d after SCI. Again, Nissl staining and HE staining showed that the area of injury site after SCI was significantly decreased, and infiltration of inflammatory cells was also reduced in SARM1^Nestin^-CKO mice at 3 d after SCI (Figure 6C-G). Finally, we performed the immunostaining of inflammatory cells by several cell markers. Interestingly, we found that the number of inflammatory cells, such as Iba1^+^ microglia, CD45^+^ immune cells and GFAP^+^ astrocytes were significantly decreased in SARM1^Nestin^-CKO mice at 3 d after SCI (Figure 6H-J, Figure S7). Taken together, these results suggested that conditional deletion of SARM1 in neurons and astrocytes reduced neuroinflammation at SCI early phase, which might promote neural regeneration.

### Conditional deletion of SARM1 in neurons and astrocytes reduced the neuroinflammation by downregulating NF-κB signaling through upregulation of HSP70 after SCI

**Figure 7:**
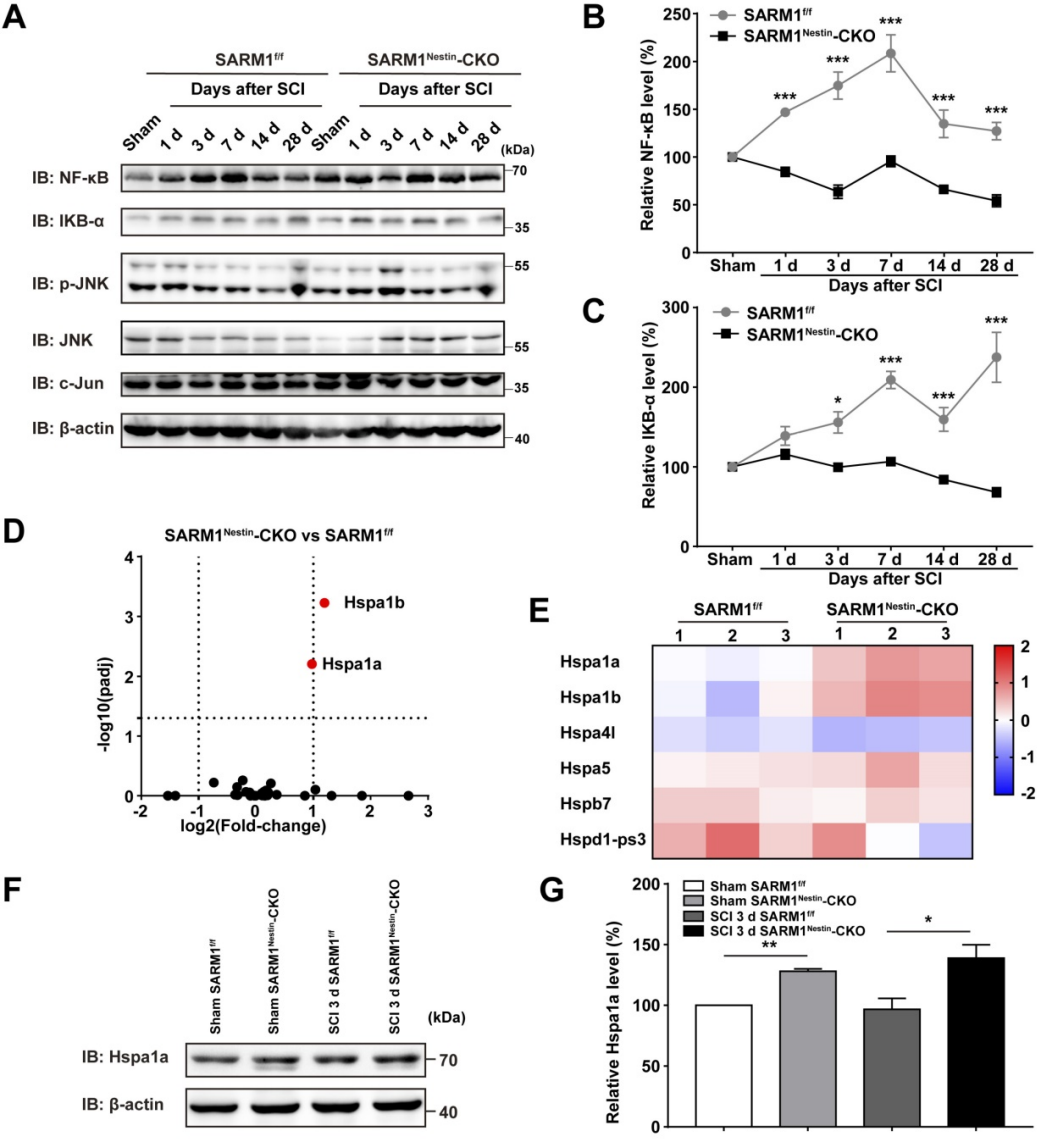
** Conditional deletion of SARM1 in neurons and astrocytes reduced the neuroinflammation through downregulation of NF-κB signaling after SCI. (A)** Western blot analysis of the expression of NF-κB, IKB-α, p-JNK, JNK and c-Jun in the spinal cords of SARM1^f/f^ and SARM1^Nestin^-CKO mice at different stages after SCI. **(B-C)** Quantitative analysis of the relative NF-κB (B) and IKB-α (C) levels as shown in (A) (two-way ANOVA (repeated measures) with Bonferroni's post-tests, *n* = 3 per group, normalized to sham group). **(D-E)** The volcano and heatmap of differentially expressed mRNAs of HSP family in the spinal cords of SARM1^f/f^ mice and SARM1^Nestin^-CKO mice. **(F)** Western blot analysis of the expression of Hspa1a in the uninjured spinal cords or injured spinal cords at 3 d after SCI of SARM1^f/f^ and SARM1^Nestin^-CKO mice. **(G)** Quantitative analysis of the relative Hspa1a levels as shown in (F) (two-tailed Student's *t*-test, *n* = 3 per group, normalized to sham group). Data were mean ± SEM. ^*^P < 0.05, ^**^P < 0.01, ^***^P < 0.001.

How did SARM1 deletion reduce the inflammation after SCI? Since NF-κB signaling is a main downstream pathway of SARM1 52, 53, we next detected the expression of probably related proteins as previously described 54, 55, such as NF-κB, IKB-α, p-JNK, JNK and c-Jun (Figure 7A). Interestingly, in the spinal cords of SARM1^f/f^ mice, NF-κB was significantly increased at all phases after SCI, and IKB-α was significantly increased at 3 d, 7 d, 14 d and 28 d after SCI, however, SARM1^Nestin^-CKO mice failed to increase the level of these proteins (Figure 7B-C). These results suggested that SARM1 knockout impaired the NF-κB signaling pathway in the spinal cords after SCI. To further support this notion, we next performed the qRT-PCR to examine the transcription level of the inflammatory factors. And the mRNA expression levels of NF-κB and IFN-α, IFN-β, IFN-γ, IL-1β, MIP-1α, TNF-α, RANTES were significantly decreased in SARM1^Nestin^-CKO mice at 3 d after SCI, compared with SARM1^f/f^ mice (Figure S8). To explore the detailed mechanism, mRNA sequencing in control spinal cords of SARM1^f/f^ and SARM1^Nestin^-CKO mice were conducted. Interestingly, the volcano and heatmap of differentially expressed mRNAs of heat shock protein (HSP) family in the spinal cords of SARM1^f/f^ mice and SARM1^Nestin^-CKO mice showed that the level of stress-inducible heat shock 70 kDa protein (HSP70) 56, 57, Hspa1, was increased in spinal cords of SARM1^Nestin^-CKO mice (Figure 7D-E). HSP70 has been reported to downregulate the NF-κB signaling pathway in several disease model 58-60. Further western blot confirmed the increased expression of Hspa1 in spinal cord tissues of SARM1^Nestin^-CKO mice 3 d after SCI (Figure 7F-G). Taken together, SARM1 deletion in neurons and astrocytes reduced the neuroinflammation after SCI, probably through downregulation of NF-κB signaling by HSP70 upregulation, which may alleviate the neuronal degeneration.

**Figure 8:**
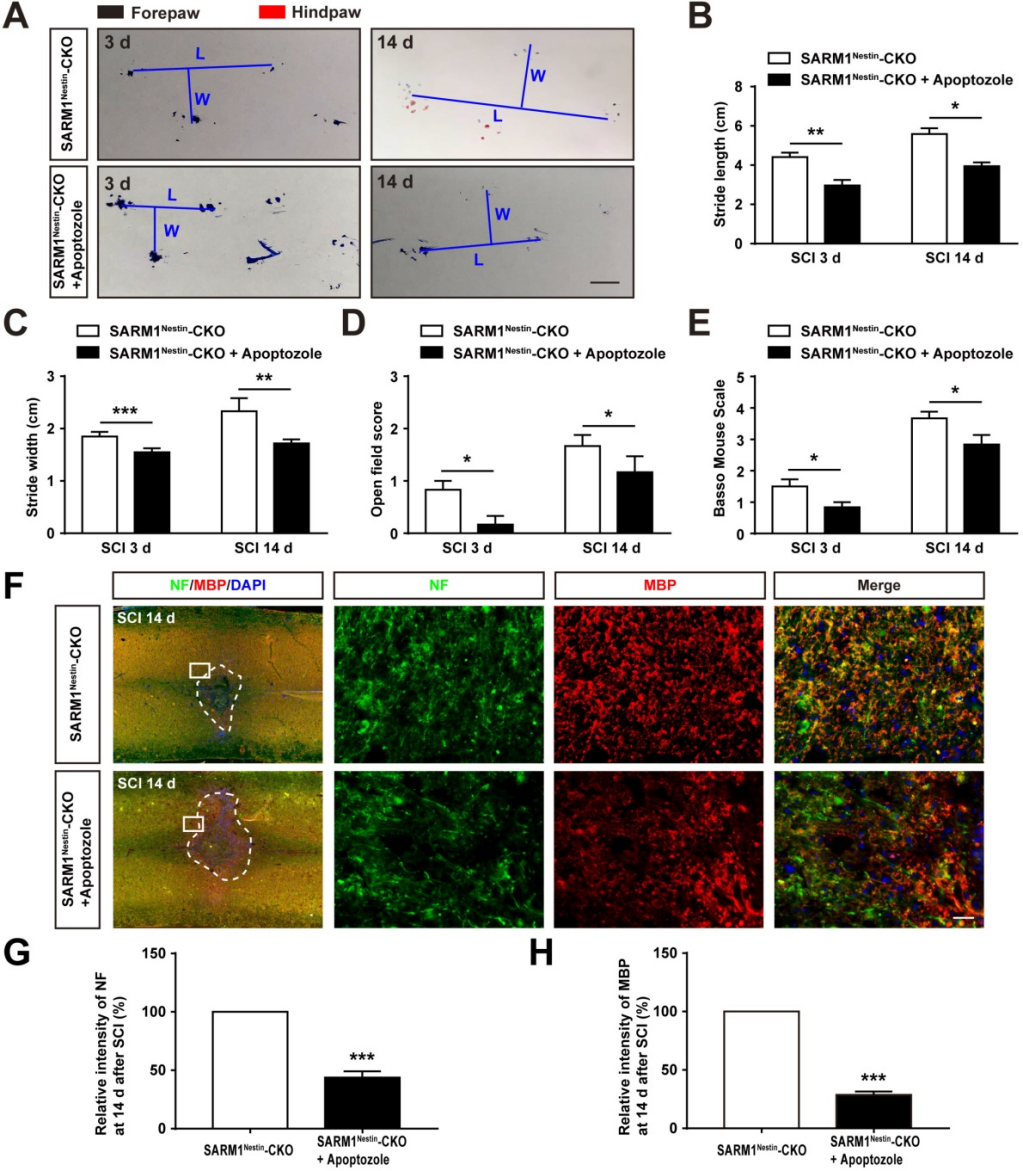
** Inhibition of HSP70 by apoptozole inhibited the recovery of behavior performance and neuronal regeneration in SARM1^Nestin^-CKO mice after SCI. (A)** Representative footprint images of SARM1^Nestin^-CKO mice with or without apoptozole treatment at 3 d and 14 d after SCI. **(B-C)** Quantitative footprint analysis of stride length (B) and stride width (C) in footprint behavioral assay of SARM1^Nestin^-CKO mice with or without apoptozole treatment at 3 d and 14 d after SCI (*n* = 6 per group). **(D-E)** Quantitative analysis of gross voluntary movement in open-field walking (D, *n* = 6 per group) and BMS scoring (E, *n* = 6 per group) of SARM1^Nestin^-CKO mice with or without apoptozole treatment at 3 d and 14 d after SCI. **(F)** Double immunostaining analysis of NF (green) and MBP (red) in the spinal cords of SARM1^Nestin^-CKO mice with or without apoptozole treatment at 14 d after SCI. **(G-H)** Quantitative analysis of the intensity of NF or MBP as shown in (F) (*n* = 6 per group, normalized to SARM1^Nestin^-CKO mice without apoptozole treatment group). Dashed lines indicated the outline of the injury sites. Images of selected regions (rectangles) in (F) were shown at higher magnification. Scale bars, 1 cm (A), 20 µm (F). Data were mean ± SEM. Two-tailed Student's *t*-test,^ *^P < 0.05, ^**^P < 0.01, ^***^P < 0.001.

**Figure 9:**
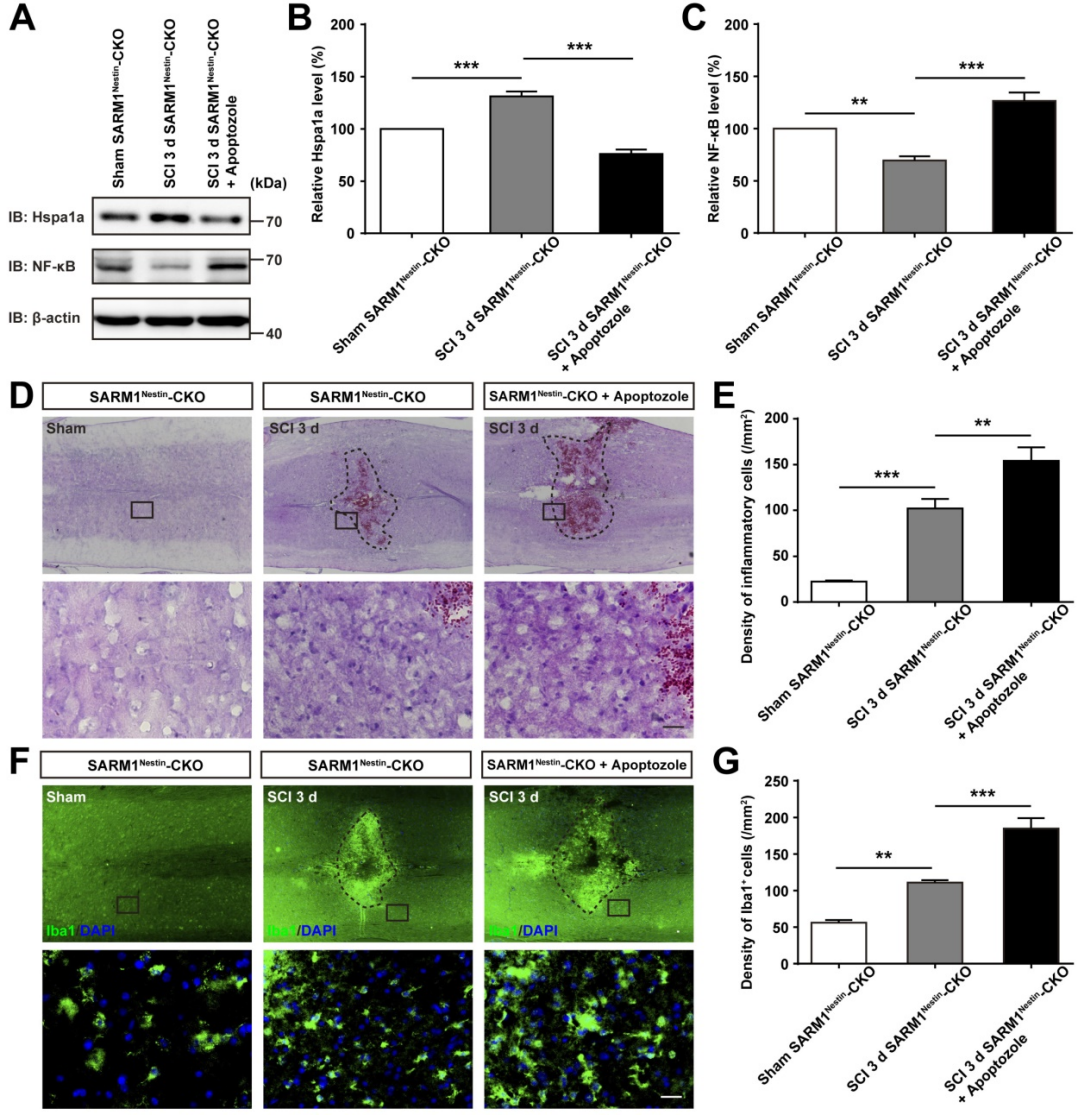
** Inhibition of HSP70 by apoptozole promoted neuroinflammation at SCI early phase. (A)** Western blot analysis of the expression of Hspa1a and NF-κB in the uninjured spinal cords from SARM1^Nestin^-CKO mice or injured spinal cords at 3 d after SCI from control or apoptozole treated SARM1^Nestin^-CKO mice.** (B-C)** Quantitative analysis of the relative Hspa1a (B) and NF-κB (C) levels as shown in (A) (*n* = 6 per group, normalized to Sham SARM1^Nestin^-CKO mice group).** (D)** HE staining images showing the inflammatory infiltration of the uninjured spinal cords from SARM1^Nestin^-CKO mice or injured spinal cords at 3 d after SCI from control or apoptozole treated SARM1^Nestin^-CKO mice.** (E)** Quantitative analysis of the density of inflammatory cells in the spinal cords as shown in (D) (*n* = 6 per group). **(F)** Immunostaining analysis of Iba1 (green) in the uninjured spinal cords from SARM1^Nestin^-CKO mice or injured spinal cords at 3 d after SCI from control or apoptozole treated SARM1^Nestin^-CKO mice. **(G)** Quantitative analysis of the density of Iba1^+^ cells as shown in (F) (*n* = 6 per group). Dashed lines indicated the outline of the injury sites. Images of selected regions (rectangles) in (D) and (F) were shown at higher magnification. Scale bars, 20 µm. Data were mean ± SEM. Two-tailed Student's *t*-test, ^**^P < 0.01, ^***^P < 0.001.

To further strengthen the relationship between HSP70 and NF-κB based on the SARM1^Nestin^-CKO mice after SCI, apoptozole, an inhibitor of HSP70 was used. As expected, behavioral assays such as footprint assays showed that apoptozole treatment significantly inhibited the functional recovery in SARM1^Nestin^-CKO mice after SCI, compared with control mice (Figure 8A-E), and immunostaining of MBP and NF showed apoptozole treatment promoted the neuronal degeneration in injury sites of SARM1^Nestin^-CKO mice after SCI (Figure 8F-H). Furthermore, western blot showed that apoptozole treatment partially rescued the decrease of NF-κB expression by SARM1 deletion in the spinal cords after SCI (Figure 9A-C). Also, HE staining (Figure 9D-E) and immunostaining of Iba1 (Figure 9F-G) showed that apoptozole treatment increased the inflammatory cells in injury sites of SARM1^Nestin^-CKO mice after SCI. Together, these findings suggested that SARM1 deletion might reduce NF-κB signaling and thereby reduce neuroinflammation by upregulating of HSP70.

### Inhibition of SARM1 by FK866 promoted the neuronal regeneration after SCI

**Figure 10:**
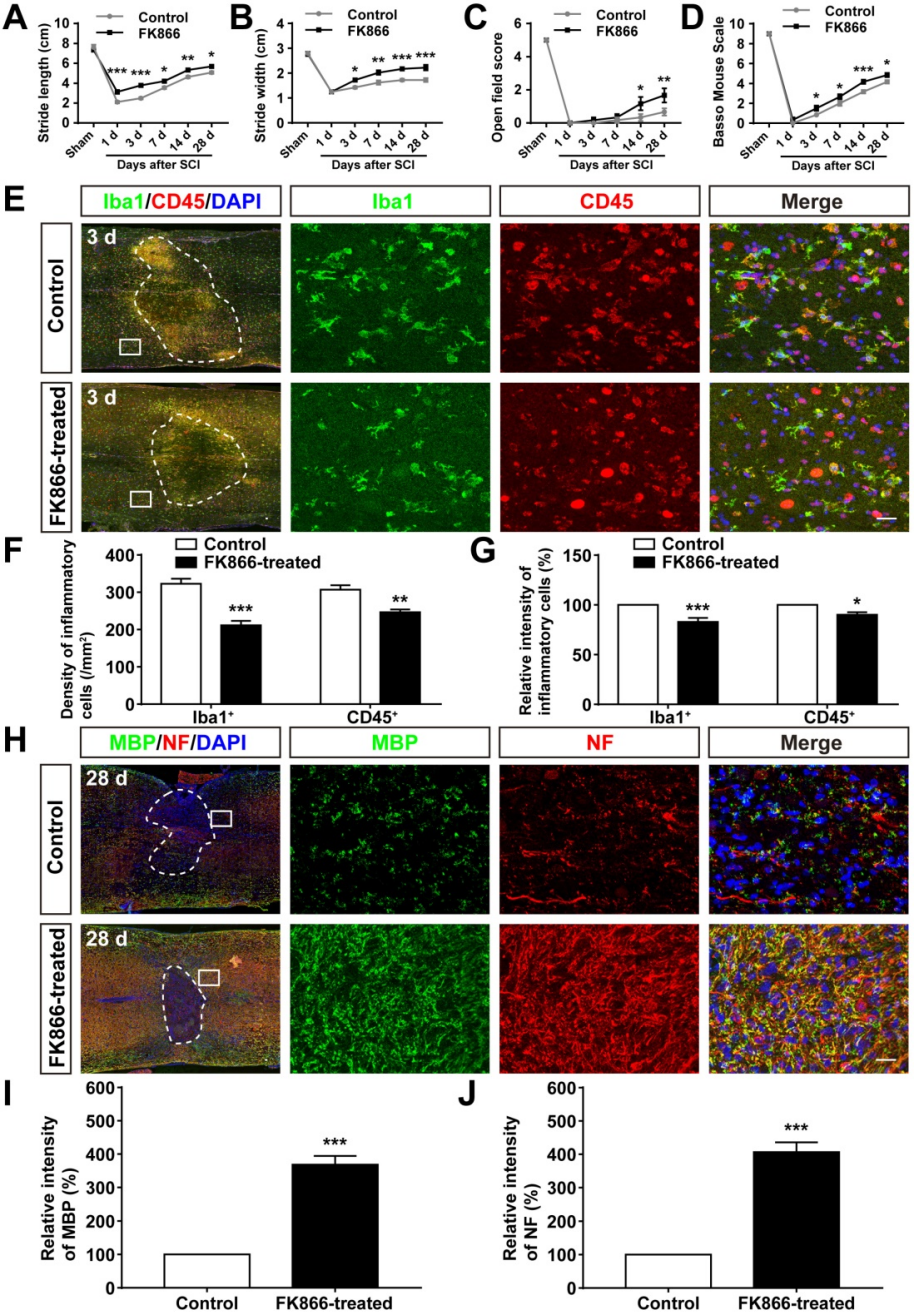
** Inhibition of SARM1 by FK866 promoted neuronal regeneration after SCI. (A-B)** Quantitative analysis of stride length (A) and width (B) in footprint assays at different stages of control (saline-treated) and FK866-treated mice after SCI (*n* = 4 per group). **(C-D)** Quantitative analysis of gross voluntary movement in the open field test (C) and BMS scoring (D) of control and FK866-treated mice in open-field walking assays over a 28-d period after SCI (*n* = 6 per group). **(E)** Double immunostaining analysis of Iba1 (green) and CD45 (red) in the spinal cords of control and FK866-treated mice at 3 d after SCI. **(F)** Quantitative analysis of the density of Iba1^+^ cells and CD45^+^ cells as shown in (E) (*n* = 6 per group).** (G)** Quantitative analysis of the intensity of Iba1^+^ cells and CD45^+^ cells as shown in (E) (*n* = 6 per group, normalized to control mice group).** (H)** Double immunostaining analysis of MBP (green) and NF (red) in the spinal cords of control and FK866-treated at 28 d after SCI. **(I)** Quantitative analysis of the intensity of MBP as shown in (H) (*n* = 3 per group, normalized to control mice group).** (J)** Quantitative analysis of the intensity of NF as shown in (H) (*n* = 3 per group, normalized to control mice group). Dashed lines indicated the outline of the injury sites. Images of selected regions (rectangles) in (E) and (H) were shown at higher magnification. Scale bars, 20 µm (E, H). Data were mean ± SEM. Two-way ANOVA (repeated measures) with Bonferroni's post-tests (A-D), two-tailed Student's *t*-test (F-G, I-J), ^*^P < 0.05, ^**^P < 0.01, ^***^P < 0.001.

We next examined whether inhibition of SARM1 pathway promoted the neuronal regeneration after SCI. FK866, a feedback inhibitor of SARM1 that raise the levels of nicotinamide (Nam) (an intermediate of energy metabolism) in the axonal compartment 61, was applied in mice after SCI. As expected, as shown in Figure 10A-D, the footprint, open field and BMS scoring analysis showed that the behavioral recovery was significantly increased in FK866-treated mice, compared with control mice. Furthermore, we found that the number of Iba1^+^ and CD45^+^ inflammatory cells was significantly decreased in FK866-treated mice at 3 d after SCI, compared with control mice (Figure 10E-G), and the intensity of MBP and NF was significantly increased in FK866-treated mice at 28 d after SCI (Figure 10H-J). Taken together, these results suggested that inhibition of SARM1 by FK866 reduced neuroinflammation and promoted neural regeneration after SCI.

## Discussion

**Figure 11:**
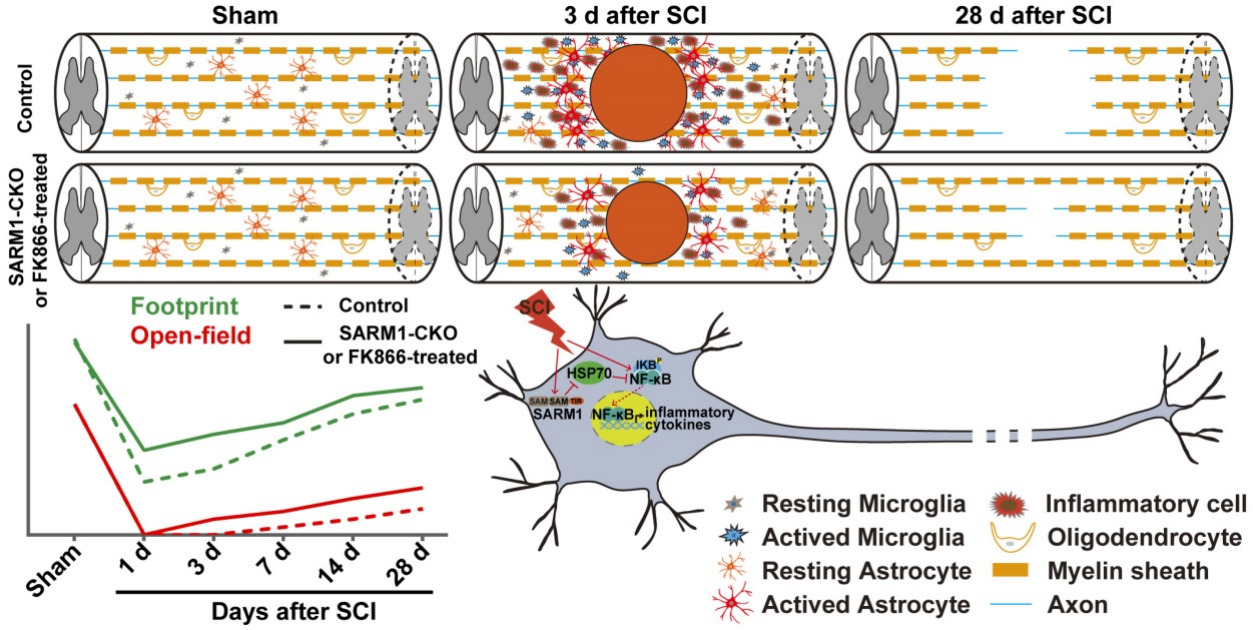
** A working model of SARM1's function after SCI.** Conditional deletion of SARM1 in neurons and astrocytes or FK866 treatment inhibited the neuroinflammation, promoted the axonal regeneration, and improved the behavioral recovery of motor function through downregulation of NF-κB signaling by HSP70 after SCI.

In this study, we provide evidence for SARM1's functions after SCI and propose a working model depicted in Figure 11. In this model, conditional deletion of SARM1 in neurons and astrocytes or FK866 treatment inhibited the neuroinflammation, promoted axonal regeneration and improved the behavioral recovery of motor function after SCI. Mechanistically, SARM1 knockout in neurons and astrocytes impaired the activation of NF-κB signal pathway through upregulation of HSP70 to inhibit the neuroinflammation after SCI, and then promoted the regeneration of neurons after SCI. Collectively, our data demonstrate that SARM1 promotes neuroinflammation and inhibits neural regeneration after SCI through NF-κB signaling pathway.

Previous researches have clarified the function and mechanism of SARM1 in neuroinflammation and axon degeneration after injuries 61-63. SARM1 is widely expressed in various tissues and cell types, and has diversity functions. Previous studies were performed based on SARM1 knockout mice, however, in our study, SARM1^flox/flox^ mice were generated, and crossed with different Cre-transgenic mice to study the functions of SARM1 in different cell types in response to different injuries. Therefore, the application of SARM1 conditional knockout mice will obtain more accurate and consolidate conclusions. Although the function and mechanism of SARM1 have been extensively studied in various kinds of injuries, its function remains unclear in spinal cord injury. Only one previous study knockdown SAMR1 by siRNA injection strategy to treat spinal cord injury, however, at the end of eight weeks, gross motor skills evaluated using the BBB score did not show any significant improvement in groups that received SARM1 siRNA injection 64. Due to limitations of siRNA, such as off-target and low transfection for neurons, the detailed role of SARM1 in spinal cord injury still remains unclear. Therefore, it is necessary to study the specific function of SARM1 in different cell types in response to spinal cord injury based on SARM1 conditional knockout mice.

SARM1 mRNA is highly expressed in the brain, lowly expressed in other tissues such as in spleen and lymph node 65. In the CNS, SARM1 mRNA is expressed in primary cultured neurons, astrocytes and microglia 65, and SARM1 protein is mainly expressed in neurons in most brain regions such as cortex, hippocampus and cerebellum 66. Consistent with these previous studies, we also found that SARM1 was highly expressed in neurons, weakly in astrocytes, but not in microglia of the spinal cords and upregulated in neurons and astrocytes after SCI. Since SARM1 has diverse functions and is expressed in several tissues 65-67, to better understand its role in neurons after SCI, conditional deletion of SARM1 mice in neurons and astrocytes, SARM1^Nestin^-CKO mice, were successfully generated in our study (Figure S4). In these mice, SARM1 was conditionally knockout in the spinal cords and cortex, hippocampus and cerebellum. Previous studies have shown that SARM1 knockout mice display defects in neuronal morphogenesis 68 and exhibit abnormal social and cognitive behaviors, but have normal locomotor activity and anxiety behaviors 69. Consistently, in our study, SARM1^Nestin^-CKO mice displayed normal development of the spinal cords and showed normal locomotor activity (Figure 2).

However, in our study, we can't exclude the role of astrocytic SARM1 in SCI, because Nestin-Cre mice express Cre recombinase in neural stem cells under the control of the Nestin promoter, thus this Cre line will knockout SARM1 in neural stem cells and their derivatives including neurons, astrocytes and oligodendrocytes. Although SARM1 was mainly expressed in neurons of spinal cords 66, and the expression levels of SARM1 were much lower in other glial cells such as astrocytes and microglia than in neurons (Figure 1 and Figure S1), however, the level of astrocytes-expressed SARM1 was increased after SCI 3 d or 14 d (Figure S3A-C), and SARM1 upregulation was not detected in microglia (Figure S3D). Therefore, SARM1 in astrocytes may also play an important role in spinal cord injury. Actually, SARM1^flox/flox^ mice were crossed with GFAP-Cre, to generate SARM1^GFAP^-CKO mice, to conditionally knockout SARM1 in astrocytes in our study (Figure S5). Our results also showed that conditional knockout SARM1 in astrocytes also improved the functional recovery after SCI based on footprint behavioral assays (Figure 4A-C), which suggest that astrocytic SARM1 may be also involved in neural regeneration after SCI. Although GFAP is commonly used as a classical marker for astrocytes in the central nervous system 70, during embryonic development, GFAP is also expressed in multipotent neural stem cells that give rise to neurons and glial cells in the brain and spinal cords 25, 71, 72. Thus, the results from SARM1^GFAP^-CKO mice after SCI can't exclude the roles of neuronal SARM1 in SCI. In the future, it is interesting to test the detailed role and mechanism of astrocytic SARM1 in SCI based on more specific astrocytic Cre mice, such as Aldh1l1-Cre mice line.

Previous studies have demonstrated that axon destruction appears in WD after SCI 73, 74. Several studies in related fields clearly demonstrated that SARM1 plays an important role in axon degeneration and in traumatic axonal injury. The severed axons from SARM1 knockout mice exhibited remarkable longer-term survival both *in vivo* and *in vitro* than wild-type mice 16. SARM1 activation triggers axon degeneration locally via NAD^+^ deprivation and a significant reduction in the number of axonal lesions early after injury was found by genetically ablating SARM1 or by FK866 treatment 19. Genetic or pharmacological interference with SARM1 signaling ameliorates early axonal pathology 75. Consistent with these previous studies, in the present study, we also found that conditional deletion of SARM1 in neurons and astrocytes reduced the area of injury site and loss of neurons after SCI, and promoted the recovery of motor functions, indicating that loss of SARM1 in neurons and astrocytes promoted the survival of neurons and delayed neuronal degeneration of the spinal cords after SCI (Figure 5). We also noted that one previous study has shown that loss of SARM1 does not suppress the degeneration of motor neurons in the SOD1^G93A^ mouse model of ALS 22. The discrepant results may be due to different animal models (ALS vs SCI). It is interesting to test how SARM1 knockout suppresses the degeneration of motor neurons in future.

Increasing studies have indicated that SARM1 regulates neuronal intrinsic immune response, white matter neuroinflammation, and prion-induced neuroinflammation 23, 62, 76. Consistent with these studies, in our study, conditional deletion of SARM1 in neurons and astrocytes reduced inflammatory infiltration and activation of microglia and astrocytes after SCI (Figure 6 and Figure S7). However, several studies have indicated that neuroinflammation is associated with WD. After CNS injury, WD is inadequate for removing inhibitory myelin debris, and most macrophages show a neurotoxic phenotype and prevent effective growth of long-distance axons 77. WD was one of the types of neuroimmune responses, which may support tissue repair 78. Our findings demonstrated that deletion of SARM1 in neurons and astrocytes contributed at least in part to the anti-neuroinflammation effect on SCI at early phase, which may promote neural regeneration at intermediate phase after SCI.

NF (neurofilament) is a marker of axonal regeneration, increased NF staining means increased axon regeneration. NF staining represents axons from the brain-spinal axon tracts. Although in our study, there was no direct evidence showed that increased MBP and NF staining in SARM1^Nestin^-CKO mice indeed the cause of improved motor functional recovery, a large body of evidence indicates that increased MBP and NF staining are associated with motor function recovery 79-82. Therefore, we speculate that increased MBP and NF staining in SARM1^Nestin^-CKO mice may be the cause of improved motor function recovery.

We also noticed that in our study, NF-κB level in the spinal cords of SARM1 conditional knockout mice was significantly higher than SARM1^f/f^ mice spinal cords under sham condition. SARM1 was originally identified as a negative regulator of the TRIF dependent TLR3 and TLR4 pathways in innate immunity 17, and some previous studies also have shown that IKB and NF-κB (p65) were upregulated in SARM1^-/-^ bone marrow-derived macrophages (BMDMs) 53. Thus, conditional knockout SARM1 in brain may somehow upregulate the expression of NF-κB level in basal level. JNK pathway was not dysregulated, which indicated that the downregulation of neuroinflammation by SARM1 conditional knockout after SCI was independent on JNK pathways. Several previous studies have shown that HSP70 exhibits an immunosuppressive activity via, e.g., downregulation of NF-κB pathway activation in disease model such as Parkinson's disease 58-60. Thus, we speculate that conditional knockout of SARM1 may inhibit the neuroinflammation through induction of HSP70, which may downregulate NF-κB pathway. Interestingly, apoptozole treatment (an inhibitor of HSP70) partially rescued the phenotypes in SARM1 conditional knockout mice after SCI (Figure 8-9). Further future studies should be performed to test detailed mechanism that how HSP70 regulates the expression of NF-κB pathway by SARM1 after SCI.

FK866, a feedback inhibitor of SARM1, has been widely used in blocking Nam consumption by inhibiting nicotinamide phosporibosyltransferase (NAMPT) 75. Previous studies have found that FK866 exerts multiple beneficial effects, including treat cancer, inflammatory diseases and neutrophil-mediated injuries 46, 83-86. Indeed, FK866 is capable of reducing the secondary inflammatory injury, and partially relieves permanent damage of SCI 61. Consistently, in our study, inhibition of SARM1 by FK866 inhibited neuroinflammation, promoted neural regeneration and promoted the recovery of behavior performance after SCI (Figure 10). Therefore, FK866 or its analogue may be effective drug to cure SCI.

In summary, our study identifies SARM1's function in SCI, SARM1 promotes neuroinflammation and inhibits neural regeneration after SCI. Therefore, discovery of inhibitors of SARM1 signaling pathway such as FK866 may be a useful drug target to treat SCI in future.

## Conclusions

In conclusion, our results based on the SARM1^Nestin^-CKO mice and SARM1^GFAP^-CKO mice indicate that SARM1-mediated prodegenerative pathway and neuroinflammation promote the pathological progress of SCI and anti-SARM1 therapeutics are viable and promising approaches for preserving neuronal function after SCI.

## Supplementary Material

Supplementary figures.

## Abbreviations

ALS: amyotrophic lateral sclerosis
BMS: Basso Mouse Scale
CKO: conditional knockout
SARM1: sterile alpha and TIR motif-containing 1
SCI: spinal cord injury
TLR: Toll-like receptor
WD: Wallerian degeneration
