# Supplementary material for: SARM1 promotes neuroinflammation and inhibits neural regeneration after spinal cord injury through NF-κB signaling
Source: Theranostics. 2021 Feb 20;11(9):4187–206. doi: 10.7150/thno.49054 (PMC7977471; doi:10.7150/thno.49054)
Supplement: Supplementary file 1 — Supplementary figures. [file thnov11p4187s1.pdf]

## Supplementary Figures and Legends

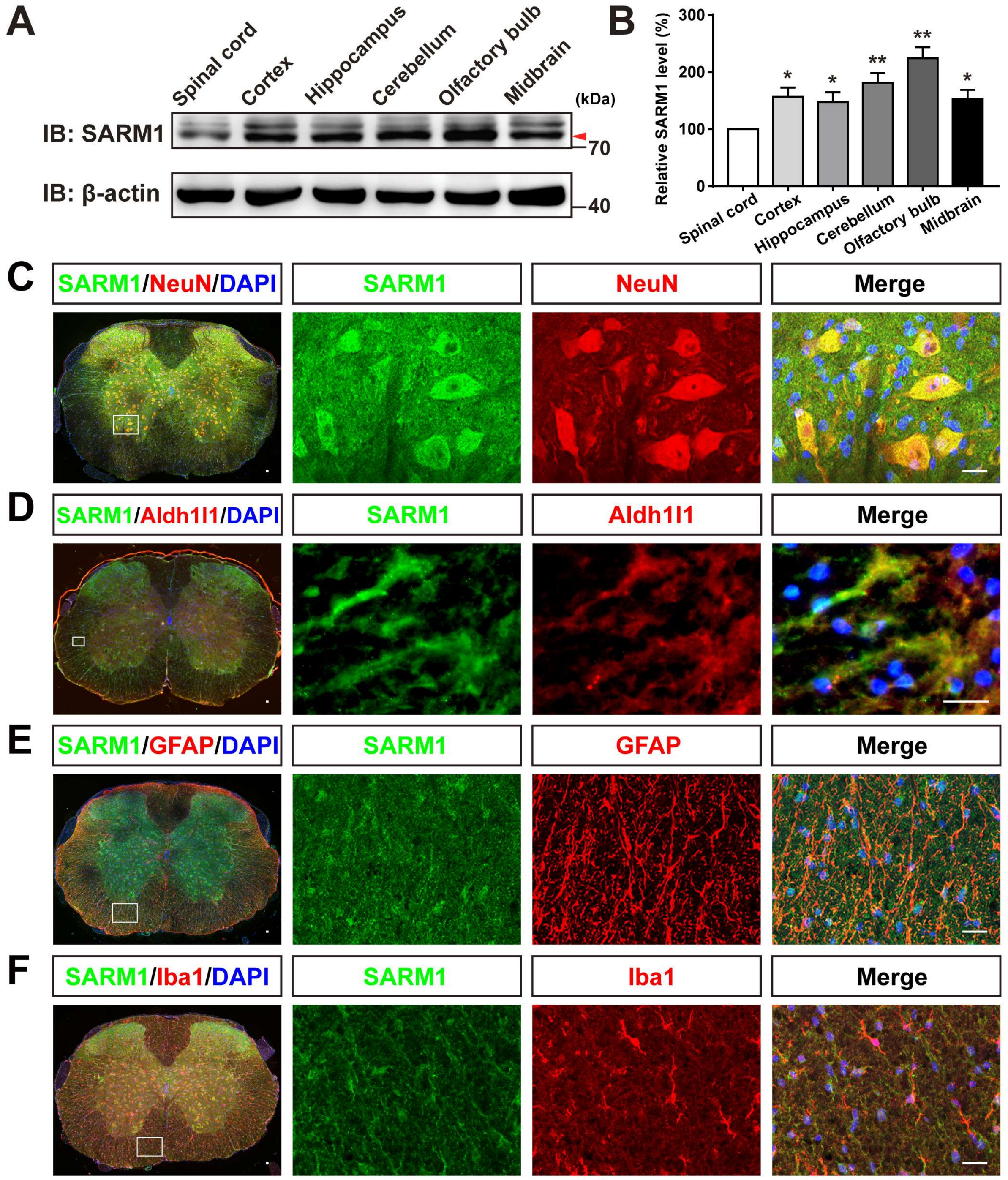


**Figure S1. Expression pattern of SARM1 in the spinal cords of mice. (A)** Western blot analysis of SARM1 expression in spinal cords and other brain regions of the mice CNS. **(B)** Quantitative analysis of the relative SARM1 levels (normalized to the spinal cord group) as shown in (A) (*n* = 5 per group). **(C-F)** Double immunostaining analysis of SARM1 (green) and NeuN (red) (C) or Aldh1l1 (red) (D) or GFAP (red) (E) or Iba1 (red) (F) in the spinal cords. Images of selected regions (rectangles) in (C-F) were shown at higher magnification. Scale bars,

20 μm. Data were mean ± SEM. Two-tailed Student's *t*-test, $^{*}P < 0.05$, $^{**}P < 0.01$.

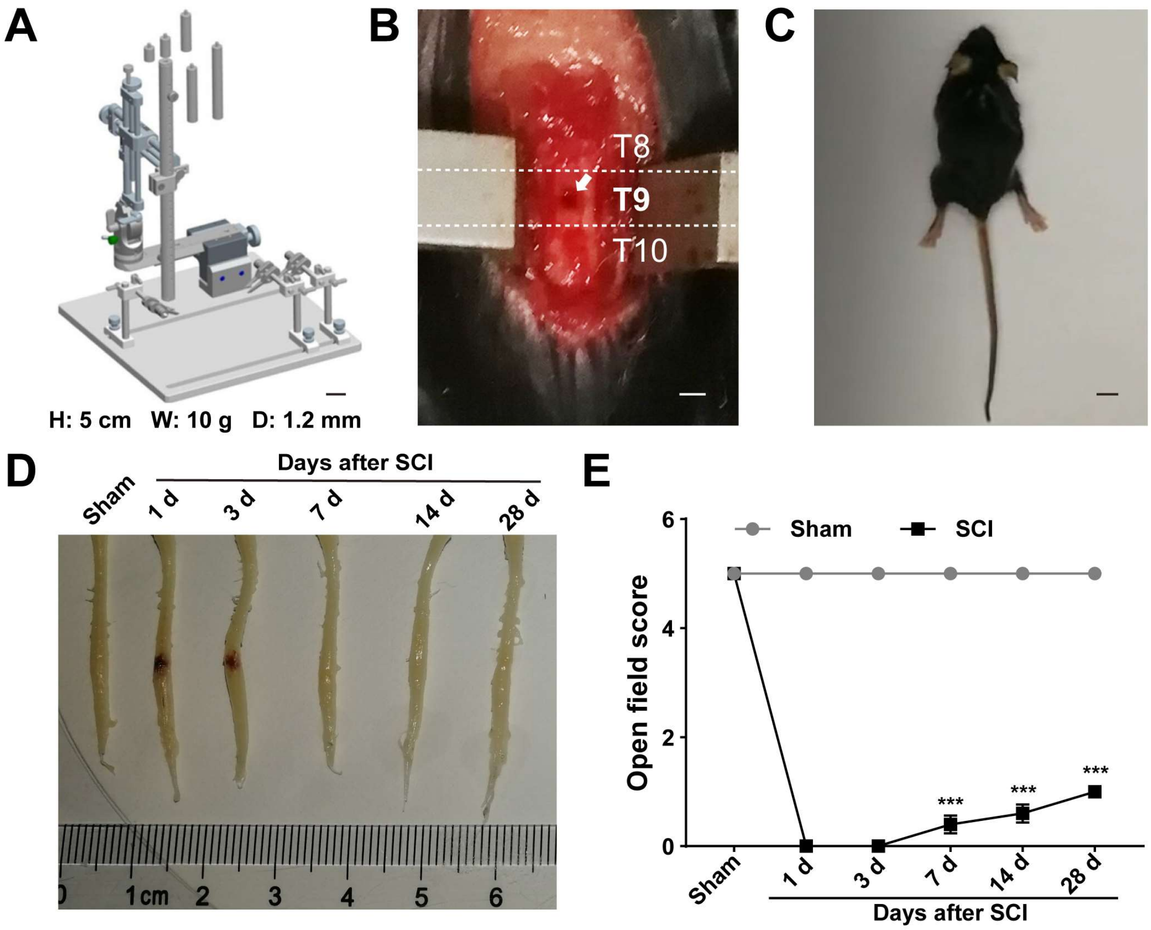


**Figure S2. Establishment of the contusion SCI mouse model. (A)** The modified Allen's contusion impactor of SCI in mice. **(B)** The impact-induced contusion (arrow) on the dorsal surface of the spinal cords. **(C)** Gross morphology of 2 M male wild-type mice at 1 d after SCI. **(D)** Representative images of the spinal cords with hematoma of wild-type mice at different stages after SCI. **(E)** Quantitative analysis of gross voluntary movement in open-field walking assays of wild-type mice over a 28-d period after SCI ($n$ = 10 per group). Scale bars, 2 cm (A), 2 mm (B), 1 cm (C). Data were mean ± SEM. Two-way ANOVA (repeated measures) with Bonferroni's post-tests, $^{***}P < 0.001$.

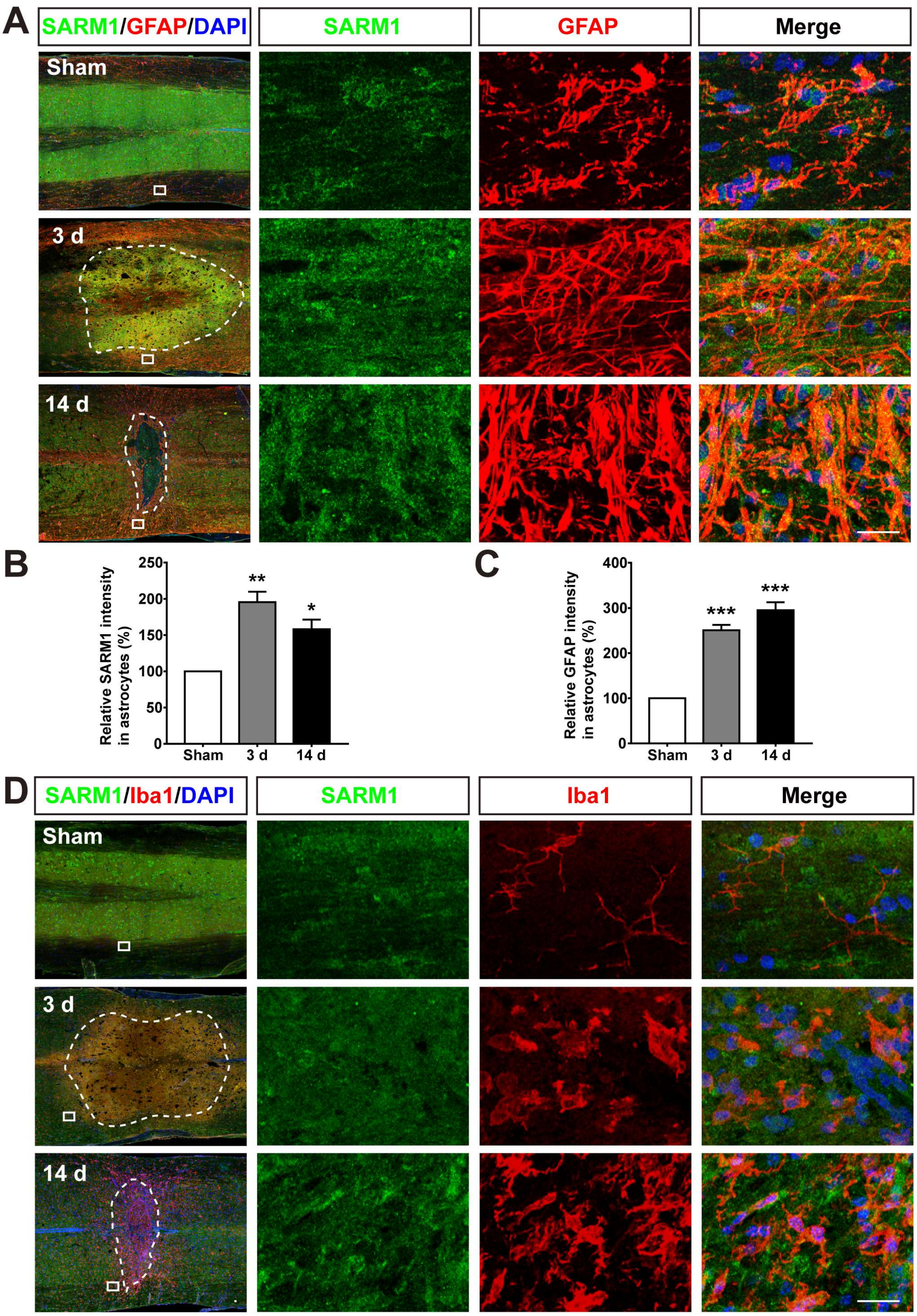


**Figure S3. SARM1 was upregulated in astrocytes, but not microglia of spinal cords at early stage after SCI. (A)** Double immunostaining analysis of SARM1 (green) and GFAP (red) in coronal sections of uninjured spinal cords and injured spinal cords at 3 d and 14 d after SCI.

**(B-C)** Quantitative analysis of the relative fluorescent intensity of SARM1 (B) and GFAP (C) level (normalized to sham group) at 3 d and 14 d after SCI as shown in (A) ($n = 3$ per group). **(D)** Double immunostaining analysis of SARM1 (green) and Iba1 (red) in coronal sections of uninjured spinal cords and injured spinal cords at 3 d and 14 d after SCI. Dashed lines indicated the outline of the injury sites. Images of selected regions (rectangles) in (A) and (D) were shown at higher magnification. Scale bars, 20 μm. Data were mean ± SEM. One-way ANOVA with Bonferroni's post-tests, $^{*}P < 0.05$, $^{**}P < 0.01$, $^{***}P < 0.001$.

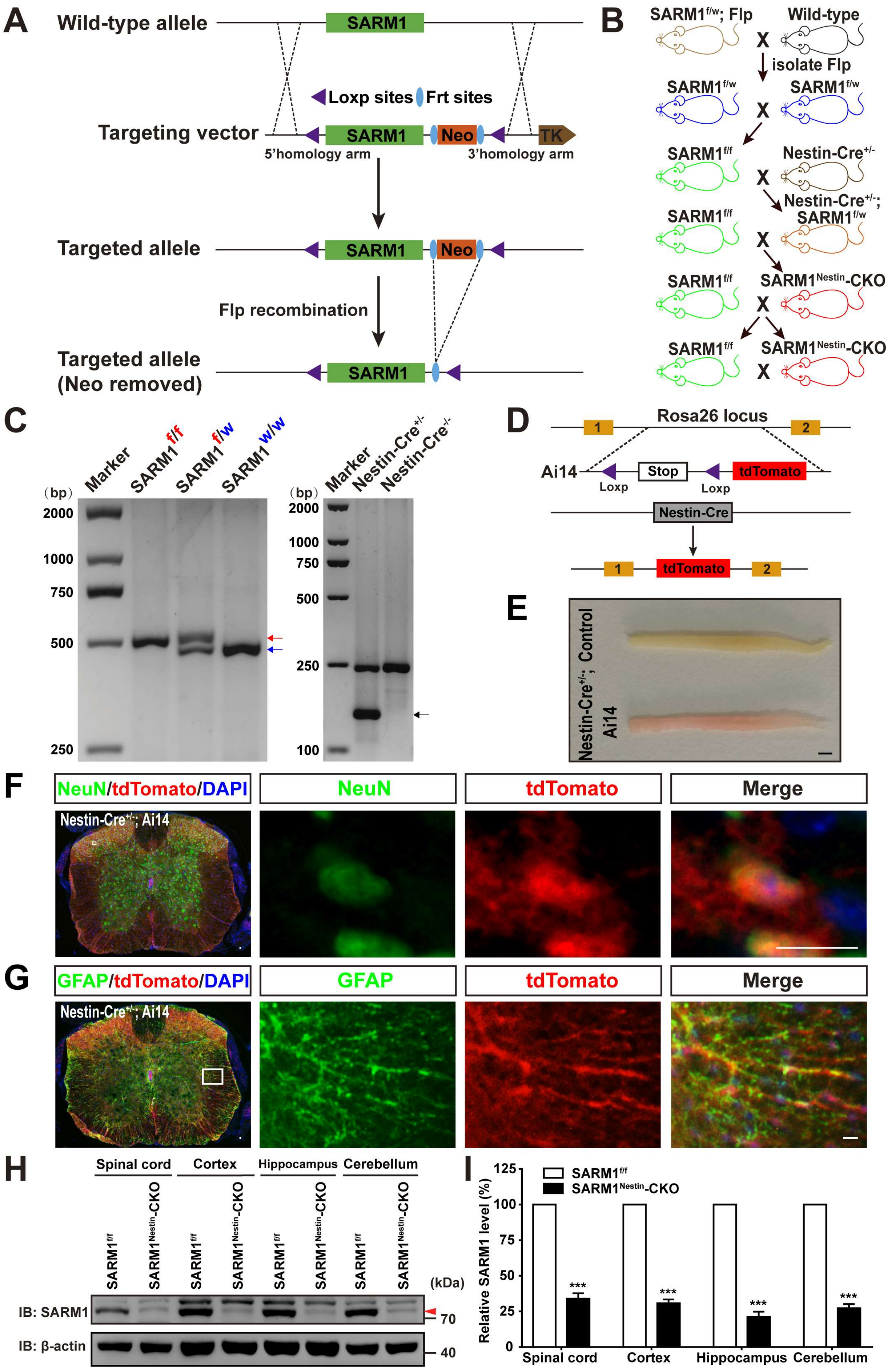
A
Wild-type allele
SARM1
Loxp sites
Frt sites
Targeting vector
SARM1
Neo
TK
5'homology arm
3'homology arm
Targeted allele
SARM1
Neo
Flp recombination
Targeted allele
(Neo removed)
SARM1
B
SARM1f/w; Flp
Wild-type
X
isolate Flp
SARM1f/w
SARM1f/w
X
SARM1f/f
Nestin-Cre+/-
X
Nestin-Cre+/-;
SARM1f/w
SARM1f/f
X
SARM1f/f
SARM1Nestin-CKO
X
SARM1f/f
SARM1Nestin-CKO
X
C
Marker
SARM1 f/f
SARM1 f/w
SARM1 w/w
(bp)
2000
1000
750
500
250
Marker
Nestin-Cre+/-
Nestin-Cre-/-
(bp)
2000
1000
750
500
250
100
D
Rosa26 locus
1
2
Ai14
Stop
tdTomato
Loxp
Loxp
Nestin-Cre
1
tdTomato
2
E
Nestin-Cre+/-; Control
Ai14
F
NeuN/tdTomato/DAPI
NeuN
tdTomato
Merge
Nestin-Cre+/-; Ai14
G
GFAP/tdTomato/DAPI
GFAP
tdTomato
Merge
Nestin-Cre+/-; Ai14
H
Spinal cord
Cortex
Hippocampus
Cerebellum
SARM1f/f
SARM1Nestin-CKO
(kDa)
IB: SARM1
70
IB: β-actin
40
I
SARM1f/f
SARM1Nestin-CKO
Relative SARM1 level (%)
125
100
75
50
25
0
***
Spinal cord
Cortex
Hippocampus
Cerebellum

**Figure S4. Generation and identification of SARM1$^{Nestin}$-CKO mice. (A-B)** Diagrams showing the strategy used to generate SARM1$^{Nestin}$-CKO mice. **(C)** Genotyping of SARM1$^{Nestin}$-CKO mice. **(D)** A diagram showing the strategy used to generate Nestin-Cre$^{+/-}$; Ai14 mice. **(E)** Images of the whole spinal cords of control (wild-type) and Nestin-Cre; Ai14 mice. **(F-G)** Immunostaining analysis of NeuN (green) (F) or GFAP (green) (G) in uninjured spinal cords of Nestin-Cre$^{+/-}$; Ai14 mice. **(H)** Western blot analysis of SARM1 expression in the spinal cords and various brain regions of 2 M male SARM1$^{f/f}$ and SARM1$^{Nestin}$-CKO mice. **(I)** Quantitative analysis of the relative SARM1 levels as shown in (H) ($n$ = 4 per group, normalized to SARM1$^{f/f}$ mice group). Images of selected regions (rectangles) in (F) and (G) were shown at higher magnification. Scale bars, 3 mm (E), 20 μm (F, G). Data were mean ± SEM. Two-tailed Student's $t$-test, $^{***}P < 0.001$.

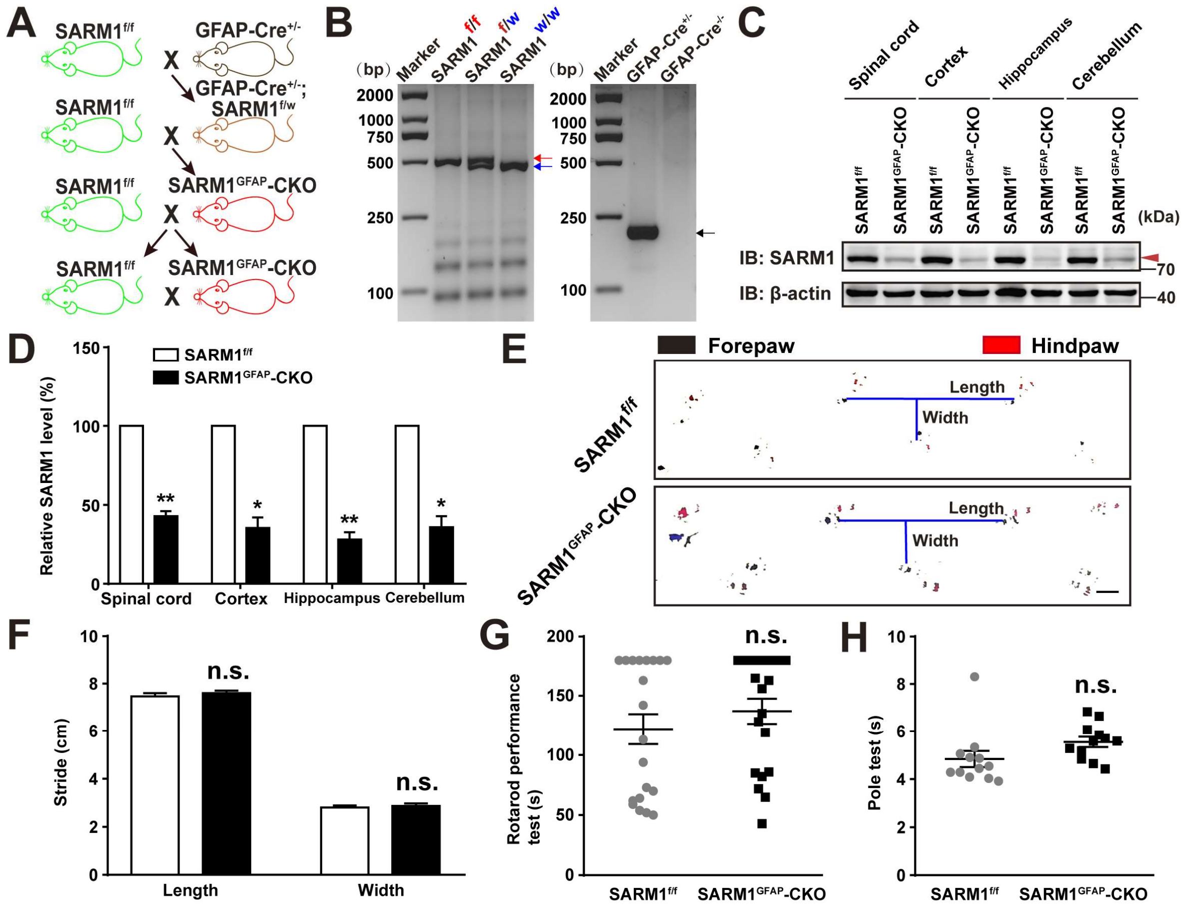


**Figure S5. Generation and identification of SARM1$^{GFAP}$-CKO mice and normal development of spinal cords of SARM1$^{GFAP}$-CKO mice. (A)** Diagrams showing the strategy used to generate SARM1$^{GFAP}$-CKO mice. **(B)** Genotyping of SARM1$^{GFAP}$-CKO mice. **(C)** Western blot analysis of SARM1 expression in the spinal cords and various brain regions of 2 M male SARM1$^{f/f}$ and SARM1$^{GFAP}$-CKO mice. **(D)** Quantitative analysis of the relative SARM1 levels as shown in (C) ($n$ = 3 per group, normalized to SARM1$^{f/f}$ mice group). **(E)** Representative footprint images of 2 M male SARM1$^{f/f}$ and SARM1$^{GFAP}$-CKO mice. **(F)** Quantitative analysis of stride length and stride width in footprint assays of 2 M male SARM1$^{f/f}$ and SARM1$^{GFAP}$-CKO mice as shown in (E) ($n$ = 6 per group). **(G)** Quantitative analysis of the time taken to fall in rotarod performance test of 2 M male SARM1$^{f/f}$ and SARM1$^{GFAP}$-CKO mice ($n$ = 12 per group)**. (H)** Quantitative analysis of the time all the four limbs took to land on in the pole test of 2 M male SARM1$^{f/f}$ and SARM1$^{GFAP}$-CKO mice ($n$ = 20 per group). Scale bars, 1 cm. Data were mean ± SEM. Two-tailed Student's $t$-test, n.s., not significant ($P > 0.05$), $^{*}P < 0.05$, $^{**}P < 0.01$.

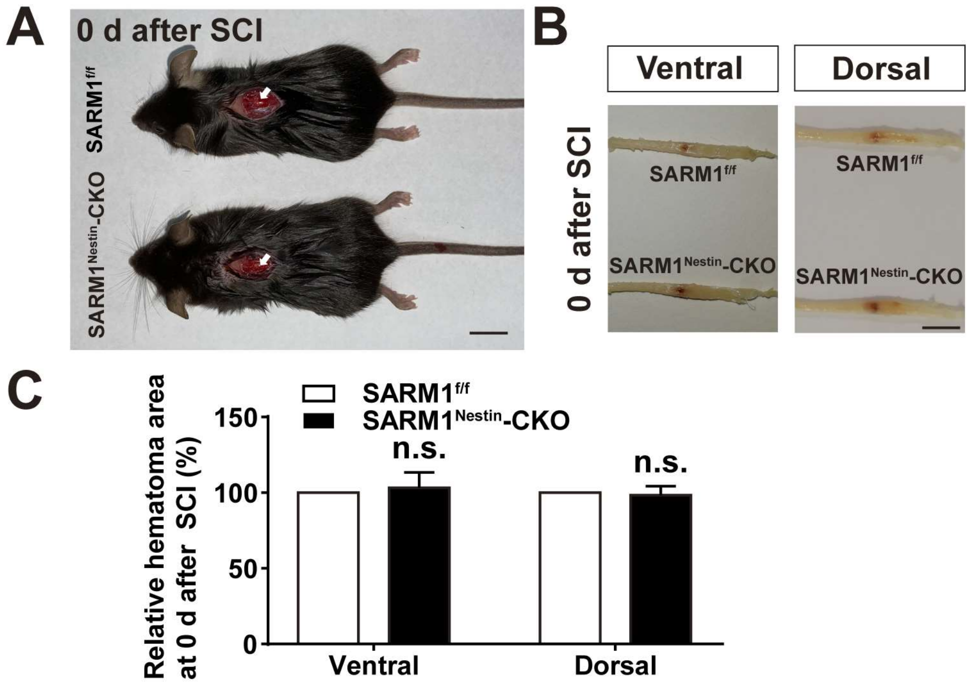


**Figure S6. The lesion areas were comparable between SARM1$^{f/f}$ and SARM1$^{Nestin}$-CKO mice. (A)** Images showed the hematoma area of 2 M male SARM1$^{f/f}$ and SARM1$^{Nestin}$-CKO mice at 0 d after SCI. **(B)** Images of the hematoma area in the whole spinal cords of 2 M male SARM1$^{f/f}$ and SARM1$^{Nestin}$-CKO mice at 0 d after SCI. **(C)** Quantitative analysis of the hematoma area at 0 d after SCI as shown in (B) ($n$ = 3 per group, normalized to SARM1$^{f/f}$ mice group). Scale bars, 1 cm. Data were mean ± SEM. Two-tailed Student's $t$-test, n.s., not significant ($P > 0.05$).

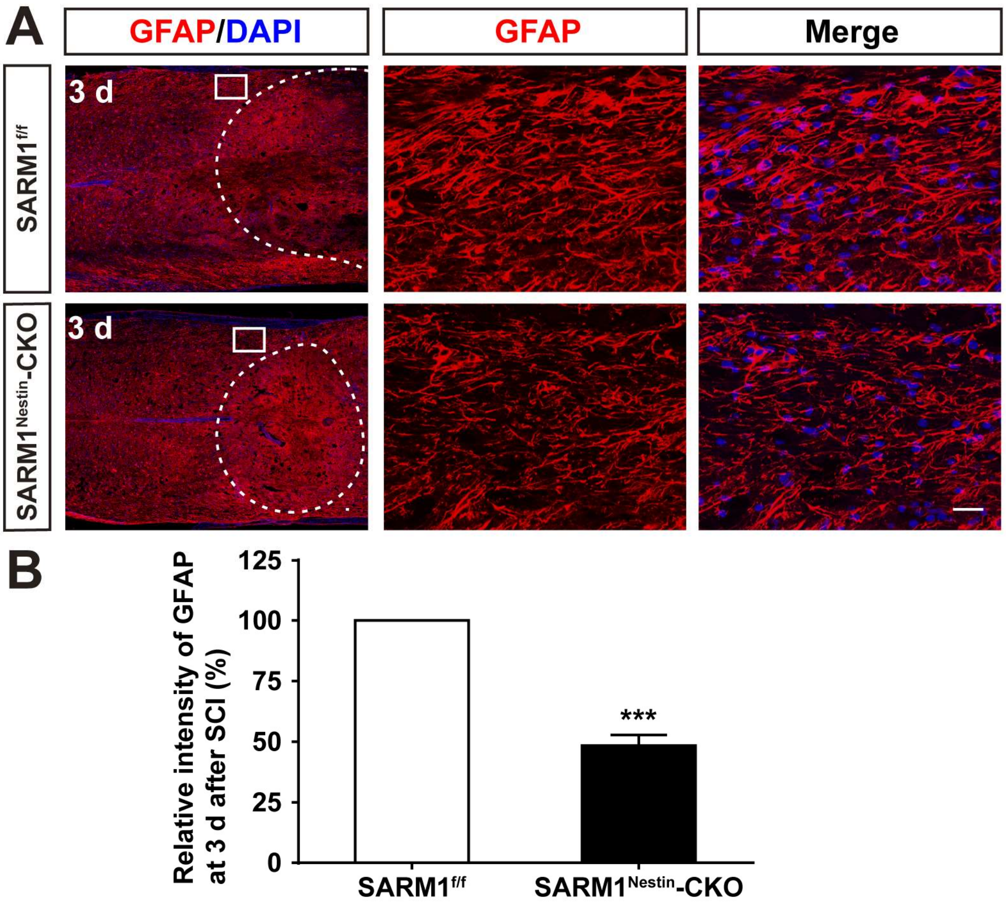


**Figure S7. The formation of glial scars was significantly decreased in SARM1$^{Nestin}$-CKO mice at 3 d after SCI. (A)** Immunostaining analysis of GFAP (red) in injured spinal cords of SARM1$^{f/f}$ and SARM1$^{Nestin}$-CKO mice at 3 d after SCI. **(B)** Quantitative analysis of the intensity of GFAP as shown in (A) ($n$ = 3 per group, normalized to SARM1$^{f/f}$ mice group). Dashed lines indicated the outline of the injury sites. Images of selected regions (rectangles) in (A) were shown at higher magnification. Scale bars, 20 μm. Data were mean ± SEM. Two-tailed Student's $t$-test, $^{***}P < 0.001$.

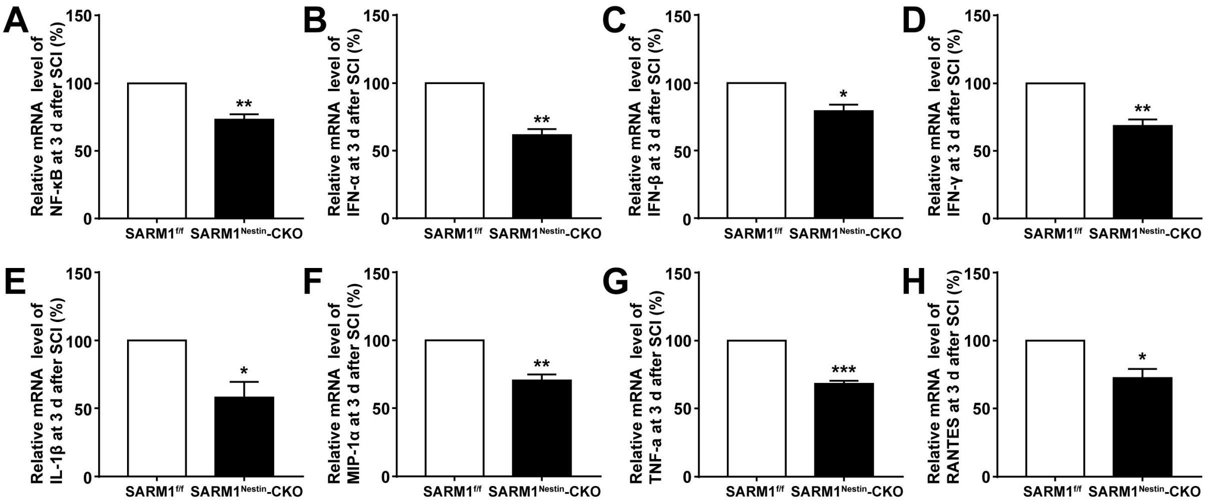


**Figure S8. Transcription level expression of inflammatory factors in injured spinal cords of SARM1$^{f/f}$ and SARM1$^{Nestin}$-CKO mice at 3 d after SCI. (A-H)** Real time PCR analysis showed the relative NF-κB (A), IFN-α (B), IFN-β (C), IFN-γ (D), IL-1β (E), MIP-1α (F), TNF-α (G) and RANTES (H) mRNA level of injured spinal cords of SARM1$^{f/f}$ and SARM1$^{Nestin}$-CKO mice at 3 d after SCI ($n = 4$ per group, normalized to SARM1$^{f/f}$ mice group). Data were mean ± SEM. Two-tailed Student's *t*-test, $^{*}P < 0.05$, $^{**}P < 0.01$, $^{***}P < 0.001$.
